# Using temperature to modify the reaction conditions and outcomes of polymers formed using transfer-dominated branching radical telomerisation (TBRT)†

**DOI:** 10.1039/d2ra06578a

**Published:** 2022-11-02

**Authors:** Sean Flynn, Oliver B. Penrhyn-Lowe, Samuel Mckeating, Stephen Wright, Sarah Lomas, Savannah R. Cassin, Pierre Chambon, Steve P. Rannard

**Affiliations:** Department of Chemistry, University of Liverpool Crown Street L69 7ZD UK srannard@liv.ac.uk; Materials Innovation Factory, University of Liverpool Crown Street L69 7ZD UK

## Abstract

Transfer-dominated Branching Radical Telomerisation (TBRT) enables the production of branched polymers with step-growth backbones using radical telomerisation chemistry. By conducting identical TBRTs over a broad temperature range, the role of temperature in telomer formation and branching has been evaluated. Elevated temperature limits telomer length, thereby allowing a >10% reduction in the amount of telogen required to produce near identical high molecular weight branched polymers.

## Abstract 2

Branched polymer synthesis by TBRT benefits from increased reaction temperatures as higher chain transfer rates lead to more efficient use of telogen, leading to reduced telogen concentrations being used to create high molecular weight polymers.

## Graphical abstract

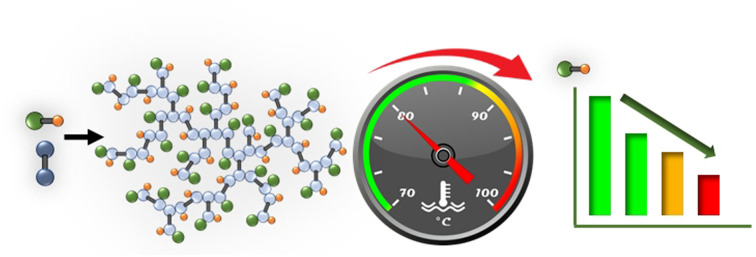

## Introduction

The formation of high molecular weight branched (co)polymer architectures *via* the radical polymerisation of multi-vinyl monomers (MVM) has challenged polymer chemists and interested researchers for decades.^1,2^ The key challenge of MVM homo- and copolymerisation is the control of the reaction of pendant vinyl groups within growing polymer chains to avoid the formation of insoluble cross-linked networks *via* unconstrained intermolecular branching reactions.^3–7^

**Fig. 1:**
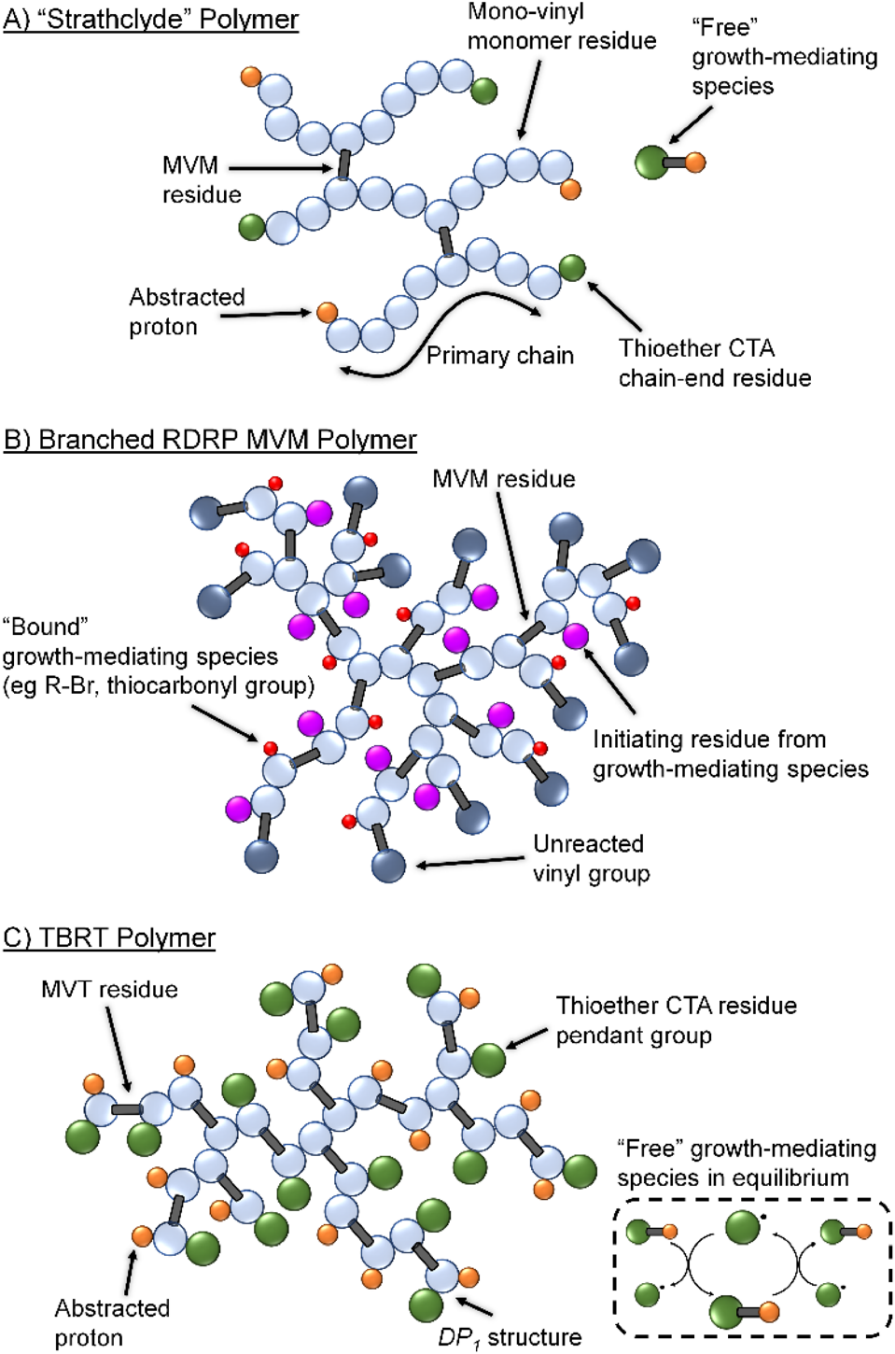
Schematic comparison of (A) statistical copolymer synthesized by multi-vinyl and mono-vinyl monomer copolymerisation under Strathclyde free radical conditions; (B) branched homopolymer synthesised from RDRP polymerisation techniques showing bound initiator/RAFT agent and numerous unreacted vinyl functional groups; and (C) branched homopolymer formed by TBRT of a multivinyl monomer (MVM) and the unbound transfer process mediating telomer formation.

Sherrington and co-workers first demonstrated the formation of branched macromolecules *via* conventional free radical copolymerisation of methyl methacrylate (MMA) with low concentrations of a bifunctional MVM, in the presence of one equivalent of a thiol-based chain transfer agent.^8^ Known as the Strathclyde methodology, this approach has proven an effective technique for the preparation of lightly branched copolymers whilst avoiding gelation,^9–11^ and has been used to create numerous novel branched polymer architectures (Fig. 1A).^12–17^ The utilisation of reversible-deactivation radical polymerisation (RDRP) techniques allowed Strathclyde strategies to be employed with additional sophistication.^18,19^ Control over the number-average degree of polymerisation (DP_*n*_) of the primary branching chains and the molar ratio of MVM to initiator allowed optimisation of branching conditions. This facilitated the preparation of high molecular weight branched copolymers with control over both the extent of branching reactions that occur and the degree of branching within the resulting polymer.

Several studies have reported detailed mechanistic studies of ‘Strathclyde’ copolymerisations and identified experimental factors which influence the polymerisation outcomes.^20,21^ Monomer concentration, MVM to initiator molar ratio, monomer chain dimensions and the statistical distribution of branching comonomers have all been identified as crucial factors in branched polymer formation.^22–26^

Control of the number and chemical nature of growing polymer chains also led to reports of MVM homopolymerisation using RDRP (Fig. 1B).^27^ However, these syntheses do not fully control the reaction and vinyl functional group conversions are limited to approximately 70 mol% even in reactions that appear to convert >95% of monomer to polymer; above 70 mol% vinyl group reaction, the unreacted pendant vinyl groups lead to gelation.^28^ Terminating such reactions before high vinyl group conversion has yielded branched polymers containing high levels of unsaturation.^29^

Several studies have aimed to further prevent/inhibit gelation by limiting propagation, namely *via* reduction of the kinetic chain length using deactivation-enhanced atom-transfer radical polymerisation.^30^ However, to the best of the authors' knowledge, homopolymerisations of MVMs have never achieved vinyl group (VG) conversions >70% using this approach.

Conventional Strathclyde polymerisations utilise thiols as chain transfer agents to limit propagation of primary chains which are predominantly formed from mono-vinyl monomer residues. Termination of primary chain-growth also limits the probability of MVM inclusion to <1 per primary chain on average. Importantly, MVM homopolymerisation using RDRP leads to the presence of chain-growth mediating species, such as initiating and chain-transfer groups, on the same growing branched polymers as pendant vinyl functionality (Fig. 1B).^30^ During vinyl group conversion, the number of unreacted pendant vinyl groups per branched macromolecule increases, as does the number of reinitiating/growth mediating species. This leads to the inevitable crosslinking and gel formation at relatively low vinyl conversions, despite the high incorporation of MVM in final polymer structures.^29^

Recently, we introduced Transfer-dominated Branching Radical Telomerisation (TBRT) as a facile and scalable synthetic strategy for the preparation of high molecular weight branched polymers and copolymers.^31,32^ TBRT utilises radical telomerisation techniques and forms polymer backbones that are analogous to step-growth chemistries (Fig. 1C). Within a conventional telomerisation, a telogen reacts with an unsaturated taxogen to form a telomer, with the propagation between taxogens limited to <5 units. Within TBRT, we utilise radical telomerisation concepts to react multi-vinyl taxogens (MVT) to high vinyl group consumption (>99%), using a high concentration of thiol-based telogens.^31^ By establishing a dominant transfer equilibrium between thiol and thiyl radical, that is independent of the growing branched macromolecules (Fig. 1C), the component telomers are maintained at a DP_*n*_ < 2 and gelation is avoided.^31^ Additionally, the free thiyl radical provides a species that is able to react with the pendant vinyl groups without an associated intramolecular branching event and allowing DP_1_ structures to form (*i.e.*, the growth mediating species is not bound to growing macromolecules). Several chemical and experimental factors have been shown to influence TBRT polymerisations, including the initial molar ratio of MVT to telogen ([MVT]_0_/[Tel]_0_), MVT dimensions and the telogen chain transfer coefficient (*C*_T_).^32–34^

**Fig. 2:**
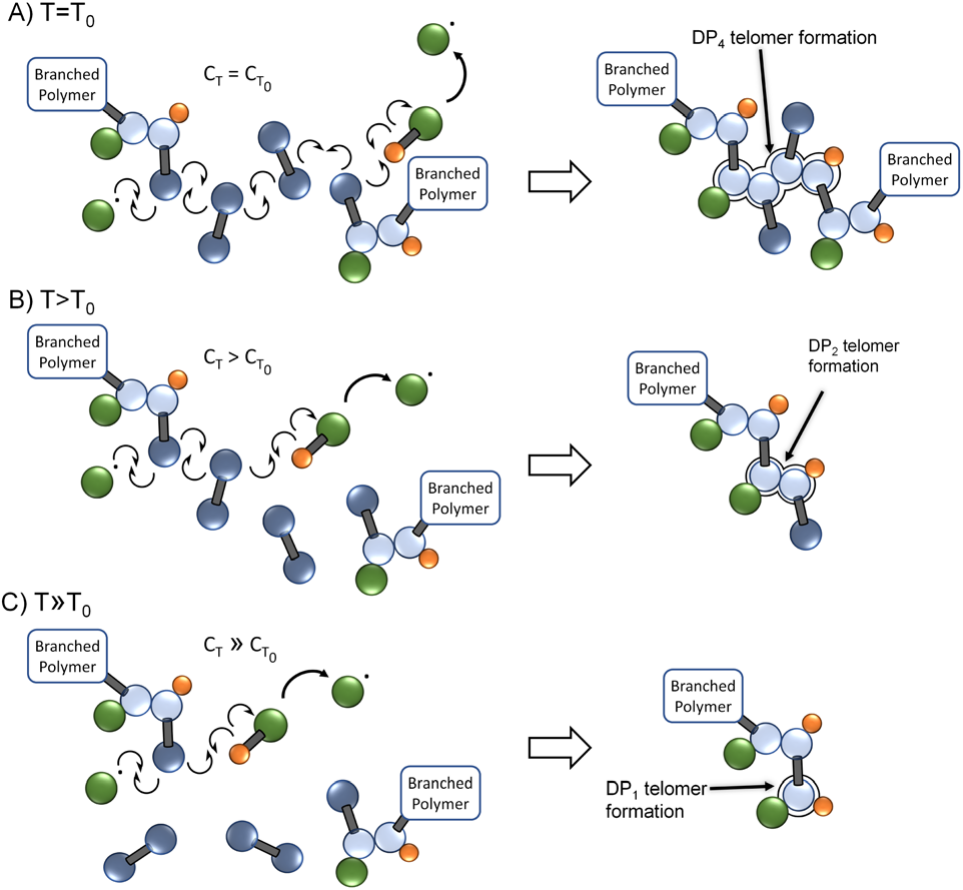
Schematic comparison of TBRT reactions occurring at (A) relatively low reaction temperature *T*_0_, (B) reaction temperature >*T*_0_, and (C) reaction temperature ≫*T*_0_. With a higher rise in chain transfer relative to propagation at higher reaction temperatures, the competing reactions are balanced increasingly towards chain transfer and short telomer subunits are formed.

As the chain transfer coefficient is defined as the ratio of the chain transfer and propagation rate constants,^35^ the potential to increase chain transfer relative to vinyl group propagation *via* increased reaction temperature is available within TBRT. This has a significant potential impact within TBRT reactions as increasing chain transfer may allow control of telomer chain subunit length *via* a single telogen, Fig. 2. Elevated temperature would be expected to make shorter telomers through enhanced chain transfer, thereby offering a route to reduction in the amount of thiol telogen required for successful high molecular weight branched polymer to be formed.^36^ Here, we explore the potential role of temperature in controlling branched polymer formation under TBRT conditions.

## Results and discussion

### Establishing *C*_T_ values

To simplify our studies, and to correlate with our later TBRT polymerisations, the variation of *C*_T_ with temperature was studied using the mono-vinyl monomer ethyl methacrylate (EMA) under free radical polymerisation conditions employing 1-dodecanethiol (DDT) as a chain transfer agent (CTA), α,α′-azoisobutyronitrile (AIBN) as radical initiator and temperatures ranging from 70 °C to 100 °C. *C*_T_ values for DDT were determined using an established Mayo method by conducting free radical polymerisations of EMA in the presence of varying concentrations of DDT. Molar ratios of DDT to EMA were systemically varied from 1 : 500 to 1 : 150 ([DDT]_0_/[EMA]_0_ = 0.002 to 0.0067, ESI Table S1†) after initially determining the kinetics of the free radical polymerisation, investigating induction periods, and determining propagation rates (ESI, Fig. S1, S2 and Table S2†). All reactions were rapidly quenched with ice before 5% vinyl conversion was achieved, as determined by ^1^H NMR spectroscopy (ESI Fig. S3†). After purification by precipitation, samples were characterised using triple detection size exclusion chromatography (TD-SEC) and molecular weights were consistently seen to decrease with increasing [DDT]_0_/[EMA]_0_ values (ESI Fig. S4†). Measured values of *M*_n_ and [DDT]_0_/[EMA]_0_ were used to construct Mayo plots for the DDT-mediated FRP of EMA at varied temperatures (ESI Fig. S5, eqn (S1)†).

At all temperatures, linear correlations were observed between [DDT]_0_/[EMA]_0_ and 1/DP_*n*_ (*r*^2^ ≥ 0.983) leading to measured *C*_T_ values of 0.64, 0.72, 0.76 and 0.83 for the DDT-mediated free radical polymerisations of EMA at 70, 80, 90 and 100 °C respectively. These values correlate extremely favourably with previous reports of similar studies of MMA under similar conditions.^37^

A relatively modest increase in *C*_T_ with increasing reaction temperature was therefore established and the observed values indicate no significant drift in the reaction composition across the [DDT]_0_/[EMA]_0_ ratios studied (ESI Fig. S6†).^38^ Although these values are within the expected range for thiol-based chain transfer agents used in the free radical polymerisation of methacrylic monomers,^39–41^ the observed temperature dependence does contrast with one previous report, which indicated that the *C*_T_ of DDT in methyl methacrylate polymerisations decreased with temperature.^42^ In general, it is expected that an increase in thiol *C*_T_ values would be seen across (meth)acrylate and acrylamide monomers at elevated temperatures.^43,44^

### The impact of temperature on TBRT

**Scheme 1:**
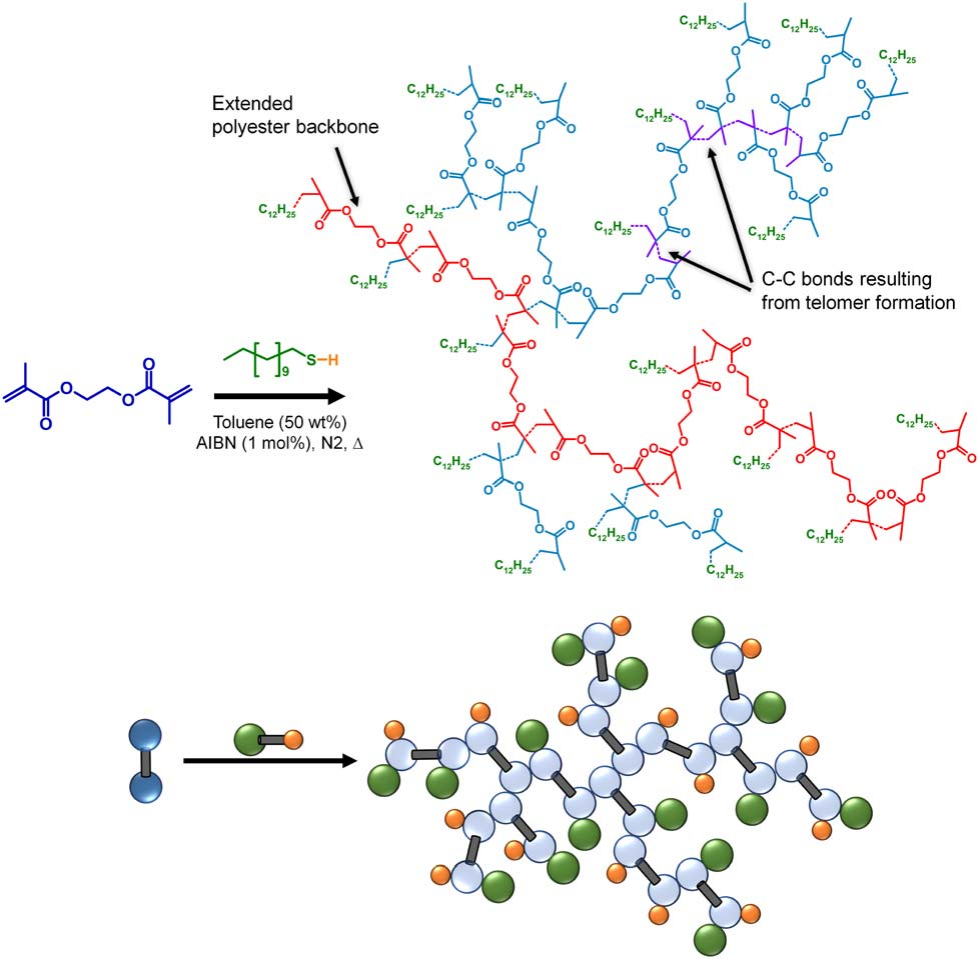
Structural and schematic representations of the formation of a branched polyester *via* TBRT of EGDMA with DDT in toluene.

TBRT relies upon chain transfer to limit the length of telomer subunits within the growing branched polymer and avoid gelation.^33^ Until this study, the impact of temperature on TBRT mechanism, and the resulting branched polymers, had not been evaluated. As stated earlier, telomerisation nomenclature describes the reaction of a telogen with an unsaturated taxogen to form a telomer. For direct correlation with the *C*_T_ evaluation described above, TBRT polymerisations were conducted using DDT as the telogen, ethylene glycol dimethacrylate (EGDMA) as the MVT, AIBN as the radical source (1 mol% w.r.t vinyl groups) and toluene as reaction solvent (Scheme 1); the same temperature range of 70–100 °C was employed. In each case, an identical initial molar ratio of EGDMA to DDT ([EGDMA]_0_/[DDT]_0_) of 0.85 was utilised with a monomer concentration ([EGDMA]_0_) of 2.3 mol dm^−3^. Initial [EGDMA]_0_/[DDT]_0_ ratios within the reaction mixture were confirmed *via*^1^H NMR analysis prior to thermal initiation (ESI Fig. S7†).

It is important to note TBRT deviates mechanistically from conventional thiol mediated free radical polymerisation of a mono-vinyl monomer. Within an ideal TBRT reaction telogen and MVT will be consumed in a 1 : 1 molar ratio at high vinyl group conversion, and significant changes in the reaction composition will be expected as the polymerisation proceeds.^31–34^ For this study, TBRT reactions were left to proceed for two hours with aliquots taken for determination of vinyl group and telogen conversion by ^1^H NMR spectroscopy and gas chromatography (GC) respectively (ESI Fig. S8, S9, Tables S3–S6†). Increased rates of both vinyl group and telogen consumption were observed with temperature; for example, TBRT conducted at 70 °C achieved near-quantitative consumption of vinyl groups after 120 minutes whereas the same conversion was achieved after just 15 minutes at 100 °C (ESI Fig. S10†).

**Fig. 3:**
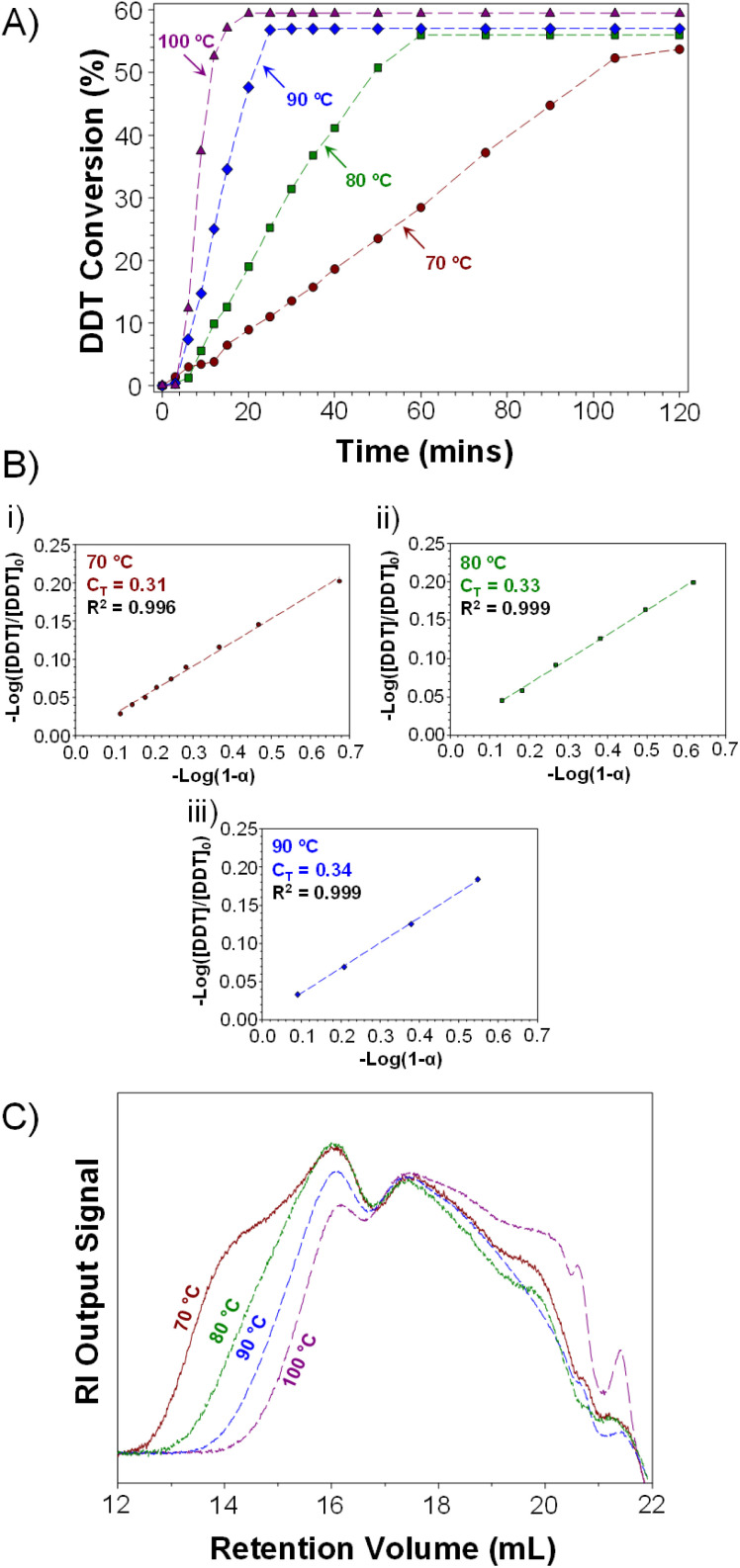
Kinetic analysis of TBRTs of EGDMA with DDT conducted at varied temperature. (A) DDT conversions as determined by GC analysis; plateau indicates completion of the TBRT reaction. (B) Calculation of DDT *C*_T_ values at (i) 70 °C, (ii) 80 °C and (iii) 90 °C *via* Smith's method and (C) overlaid RI chromatograms obtained by TD-SEC of purified polymers.

Interestingly, higher telogen conversions were achieved at elevated temperatures (Fig. 3A; note: plateau indicates end of TBRT reaction). TBRTs conducted at 70 °C, 80 °C, 90 °C and 100 °C achieved final telogen conversions of 54, 56, 57 and 59% respectively, indicating an increased overall contribution of chain transfer reactions during the synthesis of *p*(DDT–EGDMA) at higher temperatures. As TBRT is highly dominated by chain-transfer reactions, this may be significant for reaction optimisation.

Determination of vinyl group and telogen conversions enabled calculation of *C*_T_ values for DDT during the early stages of each TBRT reaction. As discussed previously, molecular weight-independent methods have been used to determine telogen *C*_T_ values during TBRT. Smith's method is of particular value in this respect, and specific relevance when telogen is being appreciably consumed.^38,41^ Both vinyl group and telogen conversion data was, therefore, used to determine *C*_T_ values for DDT during the early stages of TBRT reactions conducted across the temperature range studied (Fig. 3B); due to the speed of reactions conducted at 100 °C, sampling was not experimentally feasible at low conversion (ESI, Fig. S11†).

Surprisingly, the measured *C*_T_ values for DDT within the TBRT reactions of 0.31, 0.33 and 0.34 were lower than those observed during EMA polymerisations and appeared to show a relatively modest temperature dependence in comparison. When evaluating the conversion of DDT *vs.* vinyl groups throughout the TBRT reactions conducted at 70 °C, 80 °C and 90 °C, a near linear relationship was observed in each reaction, with a significant change in gradient seen at higher conversions (ESI, Fig. S12†). At low DDT and EGDMA conversions, the consumption of DDT is 0.301, 0.317 and 0.323 telogens per reacted vinyl group at 70 °C, 80 °C and 90 °C respectively (a 7% difference) up to approximately 70% vinyl group conversion. At higher vinyl group conversions, TBRT reactions consumed 0.465, 0.492 and 0.546 telogens per vinyl group at 70 °C, 80 °C and 90 °C respectively, representing a 17% increase in telogen consumption per vinyl group across this temperature range.

The data does imply a more dominant transfer reaction, and higher incorporation of telogen into the polymerising structures, at elevated temperatures, but importantly, the increase in chain transfer is not consistent throughout the TBRT reaction. Telogen consumption increases towards the end of the TBRT reaction (high vinyl group conversions) and this change in telogen behaviour is more evident with increasing temperature. Such behaviour would be expected to have a significant impact on the intra- and inter-molecular reactions within the branched polymerisation.^33^

Following purification by precipitation and drying *in vacuo*, the resulting branched polymers were analysed by ^1^H NMR spectroscopy (Table 1). Regardless of differences in telogen conversions achieved during TBRT, ^1^H NMR analysis showed highly comparable final molar ratios of EGDMA to telogen residues ([EGDMA]_F_/[DDT]_F_) in the purified branched polymers. [EGDMA]_F_/[DDT]_F_ ratios of 1.09, 1.10, 1.10 and 1.03 were obtained for *p*(DDT–EGDMA) syntheses conducted at 70 °C, 80 °C, 90 °C and 100 °C respectively (ESI Fig. S13†) using an [EGDMA]_0_/[DDT]_0_ ratio of 0.85 (Table 1; bold rows). Importantly, [EGDMA]_F_/[DDT]_F_ ratios represent the amount of cyclisation within the branched polymer products.^33,34^ For polymerisations conducted between 70–90 °C, approximately 9% of the EGDMA residues within the final purified and recovered *p*(DDT–EGDMA) sample are involved in cycle formation and this is remarkably consistent. For the TBRT polymer sample formed at 100 °C, this fraction appears to be considerably lower at <3%.

**Table 1:** ^1^H NMR and TD-SEC characterisation of *p*(DDT–EGDMA) polymers prepared at by TBRT at 70 °C, 80 °C, 90 °C and 100 °C using varied [EGDMA]_0_/[DDT]_0_ ratios

Temp. (°C)	^1^H NMR (CDCl_3_)			Gelation^d^	TD-SEC (THF)^e^			
	[EGDMA]_0_/[DDT]_0_^a^	Conv.^b^ (%)	[EGDMA]_F_/[DDT]_F_^c^		*M*_w_ (g mol^−1^)	*M*_n_ (g mol^−1^)	*Đ*	*α*
70 °C	0.75	>99	1.08	N	324 380	12 590	25.77	—
	0.80	>99	1.08	N	413 600	8550	48.37	0.210
	0.83	>99	1.09	N	1 732 000	40 845	42.39	0.407
	**0.85** g	**>99**	**1.09**	**N**	**2 061 000**	**52 280**	**39.43**	**0.400**
	0.88	>99	—	Y^f^	—	—	—	—
80 °C	0.75	>99	1.08	N	187 310	6700	27.95	0.349
	0.80	>99	1.09	N	253 930	9430	26.93	0.242
	0.83	>99	1.05	N	824 640	17 305	47.65	0.321
	**0.85** g	**>99**	**1.10**	**N**	**805 620**	**22 995**	**35.04**	**0.383**
	0.88	>99	1.08	N	4 207 000	313 560	13.42	0.380
	0.90	>99	1.07	N	4 847 000	321 610	15.07	0.537
	0.93	>99	—	Y^f^	—	—	—	—
90 °C	0.75	>99	1.08	N	171 620	8575	20.01	0.258
	0.80	>99	1.09	N	175 850	8160	21.56	0.271
	0.83	>99	1.08	N	522 470	14 925	35.00	0.355
	**0.85** g	**>99**	**1.10**	**N**	**424 920**	**13 515**	**31.46**	**0.375**
	0.88	>99	1.05	N	912 020	9750	94.76	0.369
	0.90	>99	1.06	N	1 532 000	18 815	81.42	0.349
	0.93	>99	1.07	N	3 394 000	154 420	21.98	0.611
	0.95	>99	—	Y^f^	—	—	—	—
100 °C	0.75	>99	1.03	N	105 960	4740	22.35	0.311
	0.80	>99	1.04	N	100 120	5545	21.56	0.308
	0.83	>99	1.08	N	224 480	6240	35.96	0.300
	**0.85** g	**>99**	**1.03**	**N**	**266 330**	**13 275**	**20.06**	**0.331**
	0.88	>99	1.10	N	498 610	9275	53.77	0.338
	0.90	>99	1.09	N	580 770	20 430	28.43	0.296
	0.93	>99	1.07	N	914 940	21 405	42.74	0.262
	0.95	>99	1.08	N	2 133 000	55 830	38.20	0.330
	0.98	>99	—	Y^f^	—	—	—	—

a Determined by ^1^H NMR spectroscopy of the polymerisation mixture prior to thermal initiation.

b Determined by ^1^H NMR spectroscopy of the polymerisation mixture after 24 hours.

c Determined by ^1^H NMR spectroscopic analysis of branched polymers following purification *via* precipitation.

d Determined by visual inspection of the polymerisation and whether branched polymer–THF solutions (<5 mg mL^−1^) were able to pass through a 0.2 μm syringe filter.

e TD-SEC analysis conducted on branched purified polymers using a THF mobile phase at a flow rate of 1 mL min^−1^.

f TBRTs that resulted in gelation could not be characterised *via*^1^H NMR and TD-SEC analysis.

g (Bold) TBRT reactions (kinetics studied in ESI Table S3–S6) compared within main discussion.

TD-SEC analysis of the *p*(DDT–EGDMA) samples generated using an [EGDMA]_0_/[DDT]_0_ ratio of 0.85 at 70–100 °C (Table 1 bold rows) showed characteristically broad molecular weight distributions for branched polymers prepared by TBRT; however, significant differences and trends in the molecular weight of the recovered branched *p*(DDT–EGDMA)s were observed (Fig. 3C, ESI Fig. S14†). A clear trend towards lower weight average molecular weights (*M*_w_) was observed as with increasing reaction temperature, with values ranging from *M*_w_ = 2 061 000 g mol^−1^ at 70 °C to 266 330 g mol^−1^ at 100 °C (Table 1). Number average molecular weight (*M*_n_) values also varied systematically from 52 280 g mol^−1^ to 13 275 g mol^−1^ across this temperature range. A concomitant decrease in dispersity was also observed, from 39.43 to 20.06, but in all cases the Mark–Houwink–Sakurada *α*-value remained at ≤0.400, indicating compact branched macromolecular architectures being formed (Table 1). The dramatic variation in *M*_w_ (approximately 10-fold) and dispersity with increasing temperature may relate directly to the observed variation in DDT consumption at higher EGDMA conversions. The increasing *C*_T_ values (Fig. 3B) and increased consumption of DDT at higher conversions (ESI Fig. S12†) strongly suggest an increased impact of chain transfer and inhibition of inter-molecular branching reactions as reaction temperature is increased.

Our previous reports have shown that variation in [MVT]_0_/[telogen]_0_ ratios may be used to increase *M*_w_ and *M*_n_;^31–34^ but the variation of TBRT outcomes at different temperatures has not been studied. A series of TBRT syntheses of *p*(DDT–EGDMA) were conducted at increasing [EGDMA]_0_/[DDT]_0_ ratios across the temperature range, until the limiting [EGDMA]_0_/[DDT]_0_ gel point ratios were identified in each case. This value represents the reaction composition where gelation cannot be effectively suppressed by the telogen and the transfer-dominant nature of TBRT no longer holds. [EGDMA]_0_/[DDT]_0_ ratios were, therefore, varied from 0.75 to 0.98 at each TBRT reaction temperature, stopping at the value where gelation occurred. In all cases, successful TBRT reactions achieved >99% vinyl group conversion (Table 1).

With increased reaction temperature, soluble branched *p*(DDT–EGDMA) was able to be formed at higher [EGDMA]_0_/[DDT]_0_ ratios with limiting gel point values being observed at 0.88, 0.90, 0.95 and 0.98 for polymerisations conducted at 70 °C, 80 °C, 90 °C and 100 °C respectively. The highest obtainable *M*_w_ values in each case varied between 2 061 000 g mol^−1^ and 4 847 000 g mol^−1^ for the recovered samples with no clear trend observed.

**Fig. 4:**
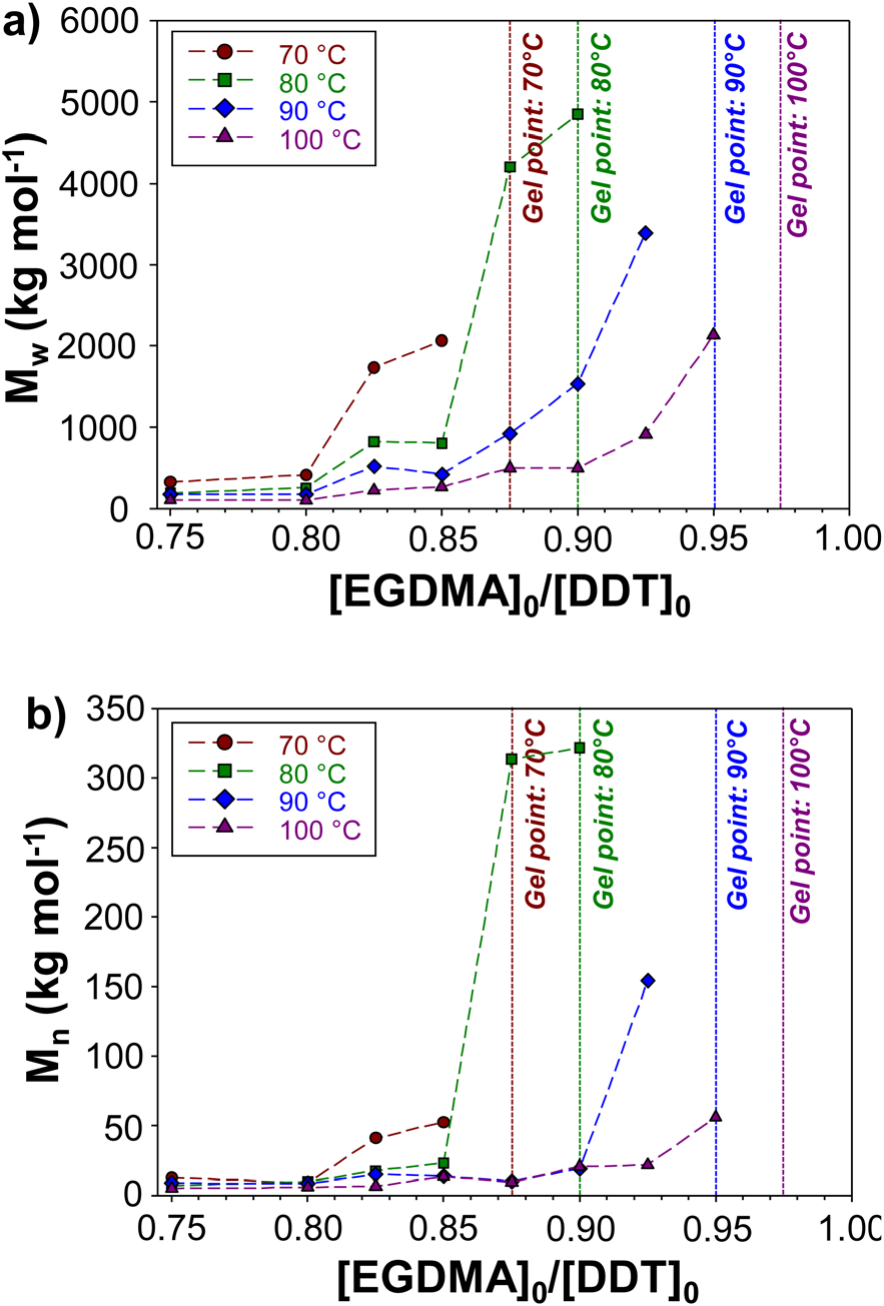
The impact of temperature on the molecular weights of branched polyesters formed by TBRT. Evolution of the (a) *M*_w_ and (b) *M*_n_ of branched polyesters generated by TBRT conducted using varied [EGDMA]_0_/[DDT]_0_ at 70, 80, 90 and 100 °C.

Within the series of polymerisations conducted at each temperature, *M*_w_ and *M*_n_ values show clear trends with increasing [EGDMA]_0_/[DDT]_0_ ratios up to the critical gel point values (Fig. 4). Across each series of reaction temperatures, *M*_w_ of polymers prepared at the same [EGDMA]_0_/[DDT]_0_ ratios decreased with polymerisation temperature, whilst *M*_w_ and *M*_n_ values increased exponentially at [EGDMA]_0_/[DDT]_0_ values approaching the critical gel point ratio (ESI, Fig. S15†). For example, at 100 °C, an increase in the [EGDMA]_0_/[DDT]_0_ ratio from 0.75 to 0.85 caused the *M*_w_ and *M*_n_ of *p*(DDT–EGDMA) to rise from 105 960 g mol^−1^ and 4740 g mol^−1^ to 266 330 g mol^−1^ and 13 275 g mol^−1^ respectively. In contrast, a similar increase from 0.90 to 0.95, caused *M*_w_ and *M*_n_ values to rise from 580 770 g mol^−1^ and 20 430 g mol^−1^ to 2 133 000 g mol^−1^ and 55 830 g mol^−1^ respectively. The relationship between the [EGDMA]_0_/[DDT]_0_ and polymer molecular weight highlights the step-growth character of the TBRT, which relies on extensive intermolecular reaction between branched polymer structures to build larger macromolecules.

Interestingly, regardless of polymerisation temperature or [EGDMA]_0_/[DDT]_0_ ratio used, *p*(DDT–EGDMA)s showed highly comparable polymer compositions, with [EGDMA]_F_/[DDT]_F_ ratios within the purified polymers varying between 1.03 and 1.10 across the whole branched polymer series (Table 1). Again, these values clearly demonstrate that temperature had minimal impact on the compositions, architecture and degree of cyclisation of the resulting branched polyesters. Differential scanning calorimetry of the highest molecular weight polyesters generated at each polymerisation temperature showed near-identical glass transition temperatures of approximately −49 °C (ESI Fig. S16†) indicating a lack of impact of polymerisation temperature on the thermal properties of the resulting branched polymers using this telogen and MVT combination.

## Conclusions

TBRT offers a new strategy for considerable branched polymer architecture synthesis and variation. The chain transfer reaction is critical to success as the balance between MVT and telogen dictates the ability to avoid gelation and the formation of soluble high molecular weight polymers. The kinetic studies described here show an expected increase in *C*_T_ with temperature, although TBRT conditions led to limited variation when compared to mono-vinyl monomer studies. During TBRT reactions, the variation in vinyl group and telogen consumption with increasing reaction temperature was highly significant, with the relative rate of telogen consumption increasing at higher vinyl group conversions at higher reaction temperatures, effectively suppressing intermolecular branching.

**Fig. 5:**
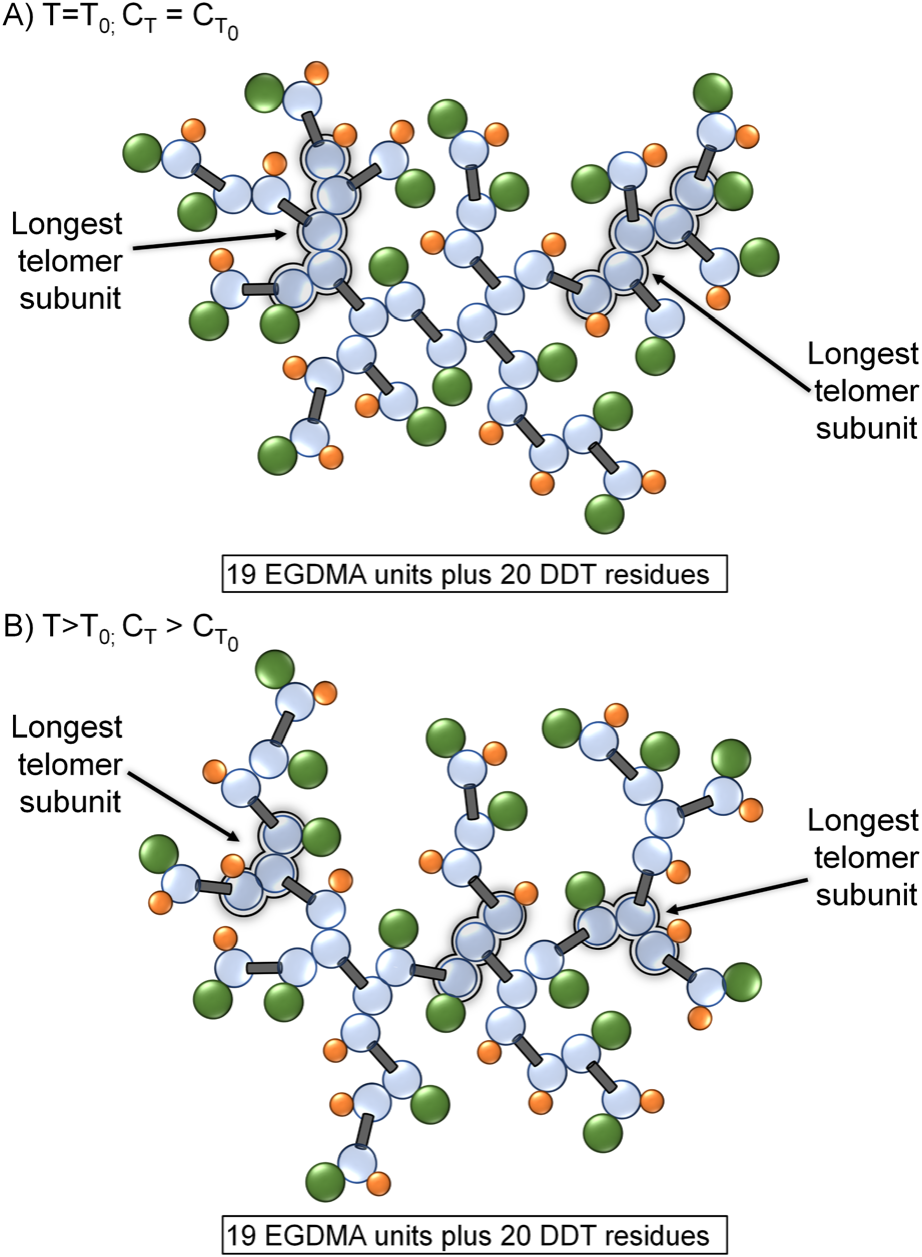
Comparative schematic representations of TBRT polymers containing identical numbers of EGDMA and DDT residues, formed at (A) relatively low temperature and *C*_T_, and (B) at higher temperature and concomitant higher *C*_T_. Telomeric substructures are reduced at higher *C*_T_ without impacting the taxogen/telogen ratio in the final polymer.

Two specific implications of CT variation at higher reaction temperatures become apparent; the first is structural. At relatively low reaction temperatures, the telomer subunits will be formed under relatively reduced chain transfer conditions and propagation will extend relatively further than the same reaction conducted at high temperature. This effect can be seen in the molecular weight data displayed in Table 1 (bold data); however, the absence of larger telomers will also lead to a branched polymer structure that has fewer structures resulting from C–C bond formation and reduced branch multiplicity (Fig. 5).

As can be seen in Fig. 5, a change in branching multiplicity does not necessarily require a different MVT/telogen ratio in the final polymer.

Secondly, as chain transfer becomes increasingly dominant over propagation at higher temperature, an increased level of molecular weight control is seen. Due to lower molecular weights being formed at higher temperatures, the [EGDMA]_0_/[DDT]_0_ ratio within the TBRT reaction may be varied above the limiting gel point values observed at lower temperatures. Within the MVT/telogen combination studied here a near 12% decrease in telogen concentration was able to be achieved by raising the reaction temperature from 70 °C to 100 °C whilst synthesising a near identical polymer. This may have significant implications for optimising further TBRT reactions.

It is important to recognise the different half-lives of AIBN across the temperature range of the studies presented here. AIBN has been reported to have a 10 hour half-life at 65 °C and this decreases to 1.2 hours at 80 °C and <15 minutes at 100 °C.^45,46^ The domination of chain transfer within TBRT, through an excess of telogen, appears to lead to control of this significant variable with initiator radicals rapidly transferred to thiol leading to a thiyl radical population that continually undergoes transfer to telogen, resulting in near identical polymer samples being generated at 70 °C and 100 °C at the respective limiting [EGDMA]_0_/[DDT]_0_ ratios. This is in line with earlier reports of reducing AIBN concentrations, where near identical polymers were recovered from TBRT reactions conducted with considerable decreases in AIBN but at fixed [EGDMA]_0_/[DDT]_0_ ratios.^31^

This study indicates that increasing the temperature of TBRT reactions may provide opportunities to enhance the atom economy of materials produced by TBRT and limit unreacted thiol within final products. Further telogen reductions may be achievable with further increases in polymerisation temperature, using high boiling point solvents such as butyl acetate (b.p. = 126 °C) and xylene (b.p. = 141 °C). Similar opportunities may be available for conventional Strathclyde polymerisations and branching polymerisations employing chain-transfer-monomers.^47^

## Conflicts of interest

SPR, SRC and PC are co-inventors on patents that protect the TBRT chemistry; these patents have been licensed to Scott Bader and form the basis of Polymer Mimetics Ltd (Company number 12598928).

## Supplementary Material

RA-012-D2RA06578A-s001
