# Supplementary material for: Using temperature to modify the reaction conditions and outcomes of polymers formed using transfer-dominated branching radical telomerisation (TBRT)
Source: RSC Adv. 2022 Nov 2;12(48):31424–31. doi: 10.1039/d2ra06578a (PMC9627727; doi:10.1039/d2ra06578a)
Supplement: RA-012-D2RA06578A-s001 [file RA-012-D2RA06578A-s001.pdf]

Electronic Supplementary Material (ESI) for RSC Advances.
This journal is © The Royal Society of Chemistry 2022

Using temperature to modify the reaction conditions and outcomes of polymers formed using Transfer-dominated Branching Radical Telomerisation (TBRT)

Sean Flynn,[a,b] Oliver B. Penrhyn-Lowe,[a,b] Samuel Mckeating,[a,b] Steve Wright,[a,b] Sarah Lomas,[a,b] Savannah R. Cassin,[a,b] Pierre Chambon,[a,b] and Steve P. Rannard*[a,b]

[a] Materials Innovation Factory, University of Liverpool, 51 Oxford Street, Liverpool, L7 3NY, UK

[b] Department of Chemistry, University of Liverpool, Crown Street, Liverpool, L69 7ZD, UK

[*] Corresponding author E-mail: srannard@liv.ac.uk

1. Materials and instrumentation

1.1 Materials

1-Dodecanethiol (DDT, 98 %), 2,2'-Azobis(2-methylpropionitrile) (AIBN, 98 %), deuterated chloroform ($CDCl_3$, 99.8 atom % D), ethylene glycol dimethacrylate (EGDMA, 98 %), and ethyl methacrylate (EMA, 99 %) were purchased from Sigma Aldrich. Hexane (analytical grade), methanol (MeOH, analytical grade 99.9 %), tetrahydrofuran (THF, HPLC-grade) and toluene (reagent grade) were purchased from Fisher. All materials were used as received unless otherwise stated.

1.2 Instrumentation

^{1}H and ^{13}C experiments were recorded using a Bruker AMX-400 MHz spectrometer; ^{1}H and ^{13}C spectra were recorded at a frequency of 400 and 100 MHz respectively. Samples were analysed in CDCl3 at ambient temperature. ^{1}H and ^{13}C chemical shifts (δ) are reported in parts per million (ppm) relative to those of tetramethyl silane (TMS).

All TD-SEC analyses were performed using a Malvern Viscotek instrument using GPCmax VE2001 autosampler, two Viscotek T6000M columns (and a guard column), a refractive index (RI) detector VE3580 and a 270 Dual Detector (light scattering and viscometer) with a mobile phase of THF at 35 °C and a flow-rate of 1 mL min^{-1}. All samples were dissolved at 5 mg mL^{-1} and passed through a 0.2 µm syringe filter prior to injection (100 µL). From TD-SEC results, the number-average molecular weight (M_n), weight-average molecular weight (M_w), polymer dispersity (Đ), molecular weight distribution and intrinsic viscosity of all polymers were estimated using Omnisec software (version 5.12). Narrow and broad polystyrene standards (Viscotek, M_w = 105 kg mol^{-1}, Đ = 1.050 and M_w = 248 kg mol^{-1}, Đ = 2.370 respectively) were used to validate the instrument operation.

Thermal analyses were conducted using a TA Instruments Discovery DSC 25 instrument equipped with a RCS90 cooling unit. The following thermal protocol was used for all DSC analyses: (1) the sample was heated to 200 °C and subjected to a 3-minute isotherm to erase its thermal history, (2) the sample was then cooled to -90 °C at a rate of -10 °C min^{-1} followed by a 30-minute isotherm. (3) glass transition behaviour was then studied during heating of the sample to 150 °C at a rate of 20 °C min^{-1}. Glass transition temperatures (T_g) were recorded using TA Instruments software, TRIOS.

2. Experimental

2.1 Determination of ethyl methacrylate free radical polymerisation kinetics

Ethyl methacrylate (20.0 g, 0.175 mol), AIBN (0.287 g, 1.75 mmol) and toluene (20.0 g, 50 wt % solids) were added to a round-bottom flask equipped with a magnetic stirrer bar. The solution was deoxygenated *via* N_2 bubbling for 30 minutes following which an aliquot was taken to aid determination of vinyl conversion *via* ^{1}H NMR spectroscopy. Samples of the mixture (*ca.* 10 mL) were transferred to individual round-bottom flasks under a N_2 atmosphere and were then purged for a further 10 minutes. Reactions were sealed and placed in separate oil baths operating at 70, 80, 90 and 100 °C. Aliquots of the reaction mixtures (~100 µL) were taken at predetermined time intervals for determination of vinyl conversion by ^{1}H NMR spectroscopy. Monomer conversions were determined *via* comparison of the integrations of chemical shifts attributed to the vinylic and methylene groups

of EMA, located at 6.10 and 4.21 ppm respectively. Crude reaction mixtures were diluted in acetone and polymers were purified *via* precipitation into hexane at ambient temperature. Polymers were dried over 48 hours *in vacuo* at 40 °C to give *p*(EMA) as a white powder.

2.2 Determination of DDT chain transfer coefficients (C_T) during free radical polymerisation of EMA at varied temperature

Chain transfer coefficients (C_T) for the free radical polymerisation of EMA in the presence of DDT were obtained *via* the Mayo method;[1] polymerisations were conducted using targeted initial molar ratios of EMA to DDT ($[EMA]_0/[DDT]_0$) of 100, 150, 200, 250, 300, 350, 400, 450 and 500. In a typical Mayo experiment, targeting an $[EMA]_0/DDT]_0$ ratio of 100, EMA (20.0 g, 0.175 mol), DDT (355 mg, 1.75 mmol), AIBN (0.287 g, 1.75 mmol) and toluene (20.0 g, 50 wt % solids) were added to a round-bottom flask equipped with a magnetic stirrer bar. The solution was deoxygenated *via* N_2 bubbling for 30 minutes. Samples of the mixture (*ca.* 10 mL) were transferred to individual round-bottom flasks under a N_2 atmosphere and were then purged for a further 10 minutes. Reactions were sealed and placed in separate oil baths operating at 70, 80, 90 and 100 °C. Polymerisations were quenched *via* rapid addition to an ice bath; this was done at time intervals that ensured monomer conversions remained below 5%. Aliquots of reaction mixtures (~100 µL) were taken for determination of vinyl group conversion by ^{1}H NMR spectroscopy. Crude reaction mixtures were purified *via* precipitation into hexane at ambient temperature. Polymers were dried over 48 hours *in vacuo* at 40 °C to give *p*(EMA) as a white powder. Molecular weight distributions of crude and purified *p*(EMA) samples were obtained using TD-SEC. C_T values were determined *via* construction and interpretation of Mayo plots.

2.3 Kinetic studies on the TBRT of EGDMA conducted at varied temperature

TBRT kinetic studies were conducted targeting an initial molar ratio of 0.850 equivalents of EGDMA per DDT ($[EGDMA]_0/[DDT]_0 = 0.850$). EGDMA (20.00 g, 101 mmol), DDT (24.02 g, 119 mmol), AIBN (332 mg, 2.02 mmol), toluene (34.02 g, 39.23 mL) and anisole (*ca.* 0.5 mL) were added to a round-bottom flask equipped with a magnetic stirrer bar. The solution was deoxygenated *via* N_2 bubbling for 30 minutes following which an aliquot was taken for determination of $[EGDMA]_0/[DDT]_0$ by ^{1}H NMR analysis. Aliquots of the mixture (*ca.* 15 mL) were transferred to individual round-bottom flasks under a N_2 atmosphere and were then purged for a further 10 minutes *via* N_2 bubbling. The flasks were sealed and placed in separate oil baths at 70, 80, 90 and 100 °C. Aliquots of reaction mixtures (~200 µL) were taken at predetermined time intervals for determination of vinyl and telogen (DDT) conversions using ^{1}H NMR spectroscopy and GC respectively. Monomer conversions were determined *via* comparison of the integrations of chemical shifts attributed to the vinylic and methylene groups of EGDMA, located at 6.10 and 4.21 ppm respectively. DDT conversions were determined *via* gas chromatography using anisole as an internal standard. TBRTs were stopped at high vinyl conversion (>99 %) by cooling to ambient temperature and exposure to air. An aliquot (~100 µL) of the reaction mixture was taken for determination of final monomer conversion *via* ^{1}H NMR analysis. Branched polymers were purified *via* precipitation from THF into methanol at ambient temperature and dried *in vacuo* at 40 °C for 48 hours. The purified samples were characterised using ^{1}H NMR spectroscopy and TD-SEC to determine final molar ratios of EGDMA and DDT structural subunits ($[EGDMA]_f/[DDT]_f$) and polymer molecular weights.

2.4 *Synthesis of hyper-branched polymers via TBRT at varied temperature*

In a typical TBRT targeting an initial ratio of 0.850 equivalents of EGDMA per DDT ($[EGDMA]_0/[DDT]_0$ = 0.850). EGDMA (10.00 g, 50.5 mmol), DDT (12.01 g, 59.4 mmol), AIBN (166 mg, 1.51 mmol) and toluene (22.18 g, 25.58 mL) were added to a round-bottom flask equipped with a magnetic stirrer bar. The solution was deoxygenated *via* N_2 bubbling for 30 minutes following which an aliquot was taken for accurate determination of $[EGDMA]_0/[DDT]_0$ by ^1H NMR analysis. Aliquots of the mixture (*ca.* 10 mL) were transferred to individual round-bottom flasks under a N_2 atmosphere and were then purged for a further 10 minutes *via* additional N_2 bubbling. The flasks were sealed and placed in separate oil baths at 70 °C, 80 °C, 90 °C and 100 °C. TBRTs were stopped after 24 hours by cooling the reaction to ambient temperature and exposure to air. Aliquots (~100 µL) of the reaction mixture was taken for determination of monomer conversion *via* ^1H NMR analysis. Hyper-branched polymers were purified *via* precipitation from THF into methanol at ambient temperature and dried *in vacuo* at 40 °C for 48 hours. Hyper-branched polymers were characterised using ^1H NMR spectroscopy and TD-SEC to determine the final molar ratios of EGDMA and DDT structural subunits ($[EGDMA]_f/[DDT]_f$) and polymer molecular weights.

3. Supplementary figures, tables and equations

Table S1 ^1H NMR and TD-SEC analysis of *p*(EMA) homopolymers generated at low conversion using varying initial molar ratios of DDT to EMA ($[DDT]_0/[EMA]_0$).

				^1H NMR ($CDCl_3$)	TD-SEC (THF)[b]				
**Entry**	**Temp. (°C)**	**$[DDT]_0/[EMA]_0$**	**$[EMA]_0/[DDT]_0$**	**Conv. (%)[a]**	**M_w (g mol^{-1})**	**M_n (g mol^{-1})**	**Đ**	**DP_n**	**$1/DP_n$**
1	70 °C	0.0067	150	3	28,315	18,625	1.52	163	0.00613
2		0.0050	200	3	33,280	21,190	1.57	186	0.00539
3		0.0040	250	4	41,270	25,510	1.62	223	0.00447
4		0.0033	300	5	43,470	27,225	1.60	239	0.00419
5		0.0029	350	3	29,735	29,735	1.65	261	0.00384
6		0.0025	400	4	53,665	32,130	1.67	281	0.00355
7		0.0022	450	3	59,850	33,515	1.79	294	0.00341
8		0.0020	500	5	71,570	35,750	2.00	313	0.00319
9	80 °C	0.0067	150	5	26,000	17,530	1.48	154	0.00651
10		0.0050	200	5	31,165	19,950	1.56	175	0.00572
11		0.0040	250	5	39,995	24,055	1.66	211	0.00474
12		0.0033	300	5	42,000	26,235	1.60	230	0.00435
13		0.0029	350	3	43,595	27,630	1.58	242	0.00413
14		0.0025	400	5	51,800	30,670	1.69	269	0.00372
15		0.0022	450	5	63,300	34,305	1.85	301	0.00333
16		0.0020	500	5	71,020	35,135	2.02	308	0.00325

[a]Determined by ^1H NMR analysis of the reaction mixture following cessation of the polymerisation. [b]Determined by TD-SEC analysis of purified polymers.

Table S1 (continued) ^{1}H NMR and TD-SEC analysis of *p*(EMA) homopolymers generated at low conversion using varying initial molar ratios of DDT to EMA ($[DDT]_0/[EMA]_0$).

				**^{1}H NMR ($CDCl_3$)**	**TD-SEC (THF)**				
**Entry**	**Temp. (°C)**	**$[DDT]_0/$ $[EMA]_0$**	**$[EMA]_0/$ $[DDT]_0$**	**Conv. (%)**	**M_w (g mol^{-1})**	**M_n (g mol^{-1})**	**Đ**	**DP_n**	**$1/DP_n$**
17		0.0067	150	4	27,425	16,874	1.63	148	0.00676
18		0.0050	200	4	33,040	20,390	1.62	179	0.00560
19		0.0040	250	3	44120	24,689	1.79	216	0.00462
20	90°C	0.0033	300	4	50,990	27,265	1.87	239	0.00419
21		0.0029	350	5	56,250	30,222	1.86	265	0.00378
22		0.0025	400	3	64,695	31,751	2.04	278	0.00359
23		0.0022	450	3	70,010	32,167	2.18	282	0.00355
24		0.0020	500	4	78,065	35,397	2.21	310	0.00322
25		0.0067	150	3	28,795	18,290	1.58	160	0.00624
26		0.0050	200	4	42,635	24,175	1.77	212	0.00472
27		0.0040	250	4	52,310	29,695	1.76	260	0.00384
28	100°C	0.0033	300	4	61,885	35,530	1.74	311	0.00321
29		0.0029	350	5	68,215	39,220	1.74	344	0.00291
30		0.0025	400	3	75,580	41,465	1.82	363	0.00275
31		0.0022	450	5	82,125	43,520	1.89	381	0.00262
32		0.0020	500	5	100,170	49,635	2.02	435	0.00230

[a]Determined by ^{1}H NMR analysis of the reaction mixture following cessation of the polymerisation. [b]Determined by TD-SEC analysis of purified polymers.

Figure S1 The impact of temperature on FRP kinetics. a) Schematic representation of the FRP of EMA. B) Kinetic plots obtained from the FRP of EMA at 70 (red circles), 80 (green squares), 90 (blue diamonds) and 100 °C (purple triangles). (i) monitoring of vinyl conversion over time and (ii) construction of first-order semi-logarithmic kinetic plots.

Table S2 ^{1}H NMR spectroscopic analysis of EMA polymerisation kinetics at varied temperature.

Entry	Temp. (°C)	Time (mins)	^{1}H NMR ($CDCl_3$)		
			Vinyl Conv. (%)[a]	$[M]_0/[M]$	$\ln[M]_0/[M]$
1	70 °C	5	1	1.01	0.01
2		10	6	1.06	0.06
3		15	10	1.10	0.10
4		20	13	1.15	0.14
5		25	16	1.19	0.17
6		30	20	1.25	0.22
7		40	25	1.34	0.29
8		50	31	1.44	0.37
9		60	37	1.58	0.46
10		75	44	1.80	0.59
11		90	51	2.02	0.71
12		120	62	2.64	0.97
13	80 °C	5	7	1.08	0.08
14		10	17	1.20	0.19
15		15	27	1.36	0.31
16		20	36	1.56	0.44
17		25	42	1.73	0.55
18		30	47	1.88	0.63
19		40	56	2.28	0.82
20		50	63	2.73	1.01
21		60	70	3.33	1.20
22		75	78	4.48	1.50
23		90	83	5.85	1.77
24		120	92	13.16	2.58
25	90 °C	5	1	1.01	0.01
26		10	6	1.06	0.06
27		15	10	1.10	0.10
28		20	13	1.15	0.14
29		25	16	1.19	0.17
30		30	20	1.25	0.22
31		40	25	1.34	0.29
32		50	31	1.44	0.37
33		60	37	1.58	0.46
34		75	44	1.80	0.59
35		90	51	2.02	0.71
36		120	62	2.64	0.97

Table S2 (continued) ^{1}H NMR spectroscopic analysis of EMA polymerisation kinetics at varied temperature.

Entry	Temp. (°C)	Time (mins)	^{1}H NMR ($CDCl_3$) Vinyl Conv. (%)[a]	$[M]_0/[M]$	$\ln[M]_0/[M]$
37	100 °C	5	7	1.08	0.08
38		10	17	1.20	0.19
39		15	27	1.36	0.31
40		20	36	1.56	0.44
41		25	42	1.73	0.55
42		30	47	1.88	0.63
43		40	56	2.28	0.82
44		50	63	2.73	1.01
45		60	70	3.33	1.20
46		75	78	4.48	1.50
47		90	83	5.85	1.77
48		120	92	13.16	2.58

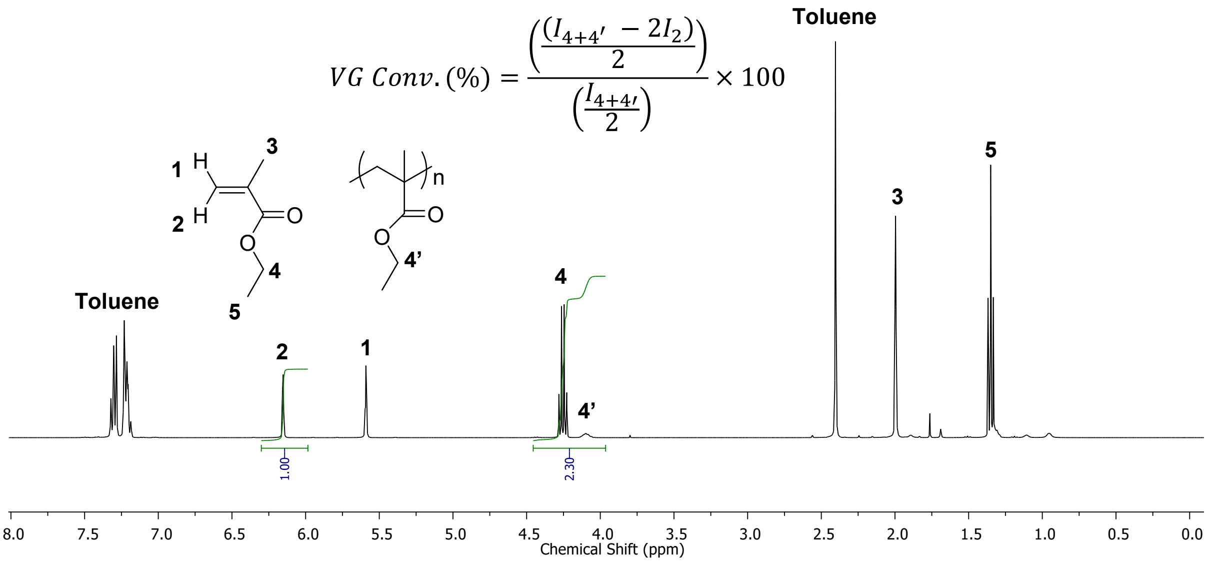

Figure S2 Exemplary determination of vinyl group (VG) conversion by ^{1}H NMR spectroscopy (400 MHz, $CDCl_3$). ^{1}H NMR spectrum obtained from the FRP of EMA in toluene (50 wt %) at 70 °C after 20 minutes. The equation provided was used to determine vinyl group conversion (13%).

$$\frac{1}{DP} = \frac{1}{DP_0} + C_T\left(\frac{[S]_0}{[M]_0}\right)$$

Equation S1

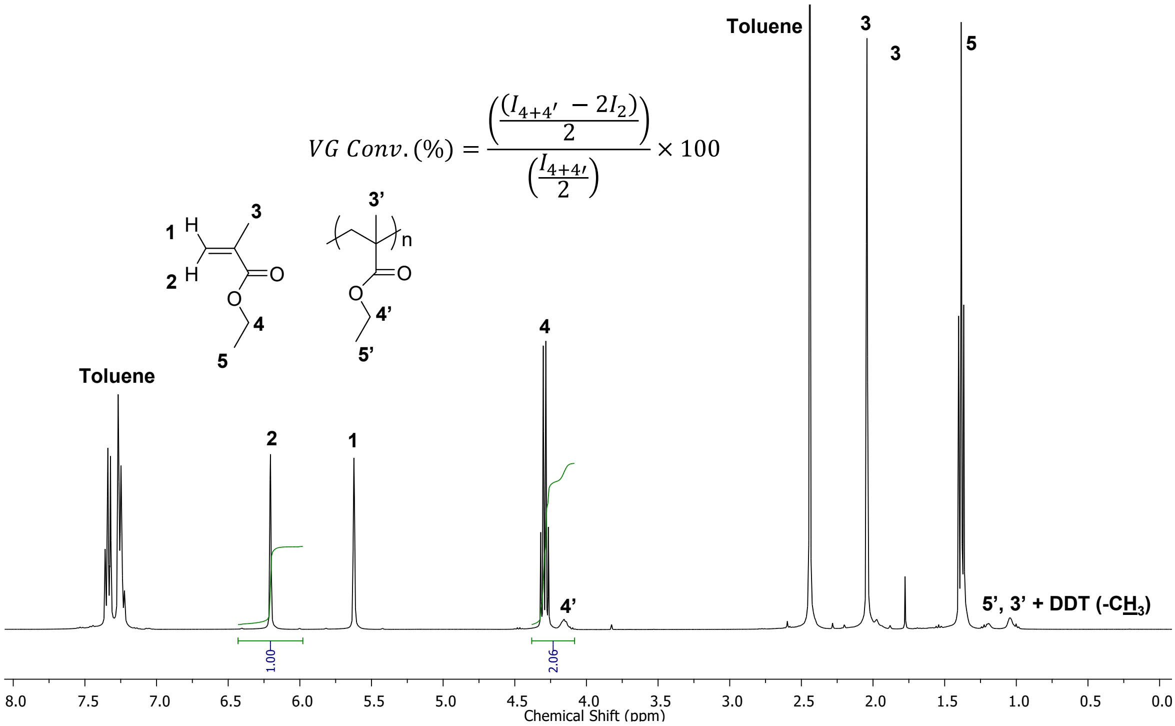

Figure S3 Exemplary determination of vinyl group (VG) conversion by ^{1}H NMR spectroscopy (400 MHz, $CDCl_3$). ^{1}H NMR spectrum obtained from the FRP of EMA in the presence of 1-dodecane thiol (DDT) at 70 °C in toluene (50 wt %). The equation provided was used to determine vinyl group conversion (3%).

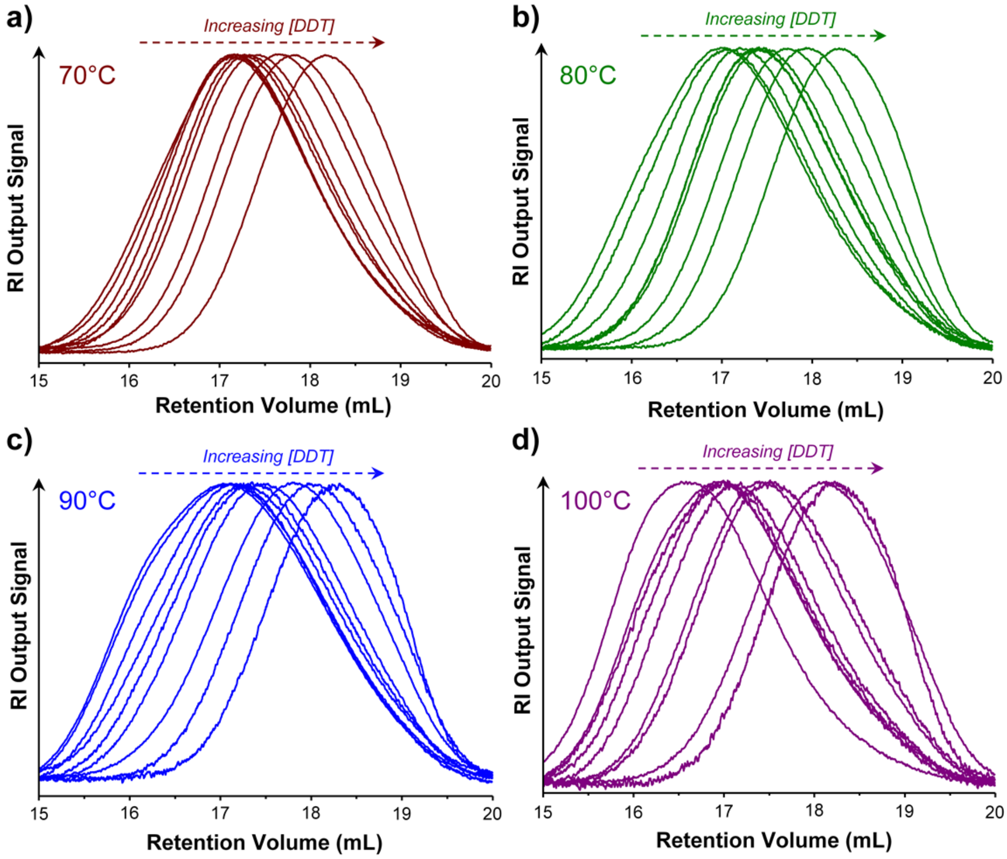

Figure S4 The impact of $[DDT]_0/[EMA]_0$ on the molecular weight distribution of *p*(EMA). Overlaid RI chromatograms obtained by TD-SEC of *p*(EMA) generated at a) 70°C, b) 80°C, c) 90°C and d) 100°C using varied $[DDT]_0/[EMA]_0$ ratios. In all cases, Mayo analyses were conducted using $[DDT]_0/[EMA]_0$ ratios of 0.0067, 0.0050, 0.0040, 0.0033, 0.0029, 0.0025, 0.0025, 0.0022, 0.0020.

Figure S5 The impact of temperature the chain transfer coefficient, C_T. a) Schematic representation of the CTA-mediated FRP of EMA. B) Mayo plots obtained at (i) 70 (red circles), (ii) 80 (green squares), (iii) 90 (blue diamonds) and (iv) 100 °C (purple triangles).

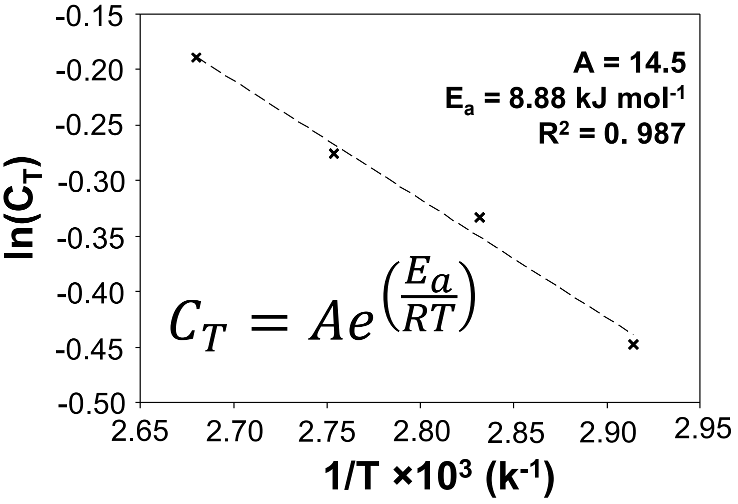

Figure S6 The impact of temperature the chain transfer coefficient, C_T. An Arrhenius plot of ln(C_T) *vs.* 1/T using C_T values obtained for DDT at temperatures varying between 70 and 100 °C.

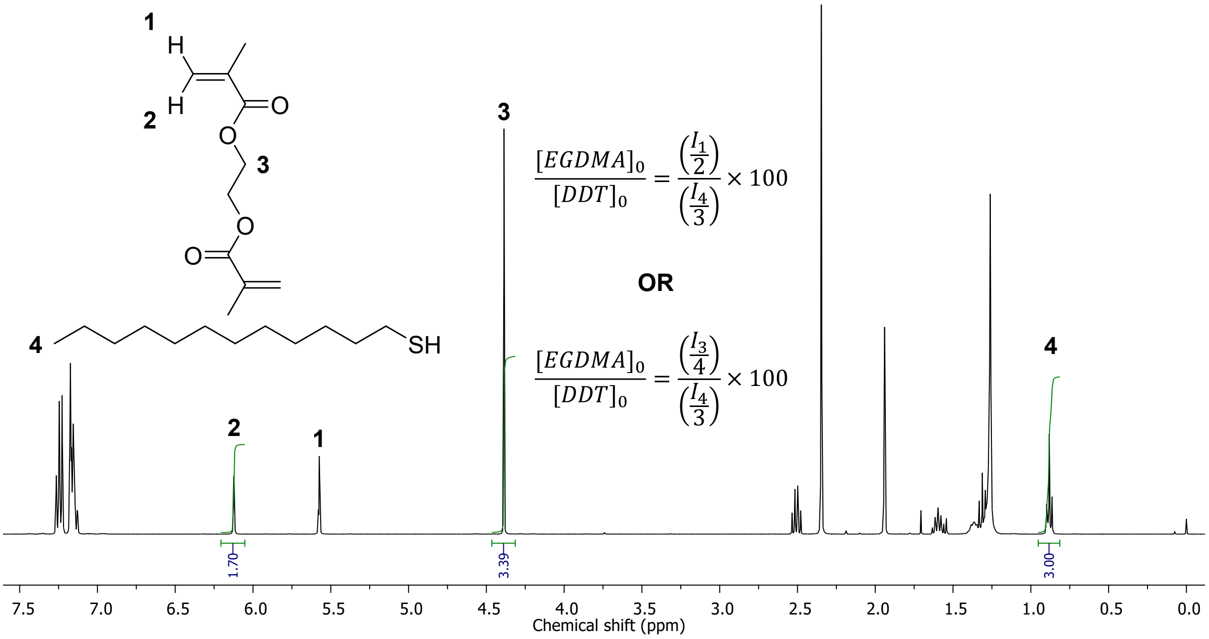

Figure S7 Determination of $[EGDMA]_0/[DDT]_0$. Exemplary ^{1}H NMR spectrum of a TBRT reaction mixture prior to initiation. The equations provided show how the intensities of EGDMA and DDT chemical shifts were used to determine the $[EGDMA]_0/[DDT]_0$ ratio ($[EGDMA]_0/[DDT]_0 = 0.85$).

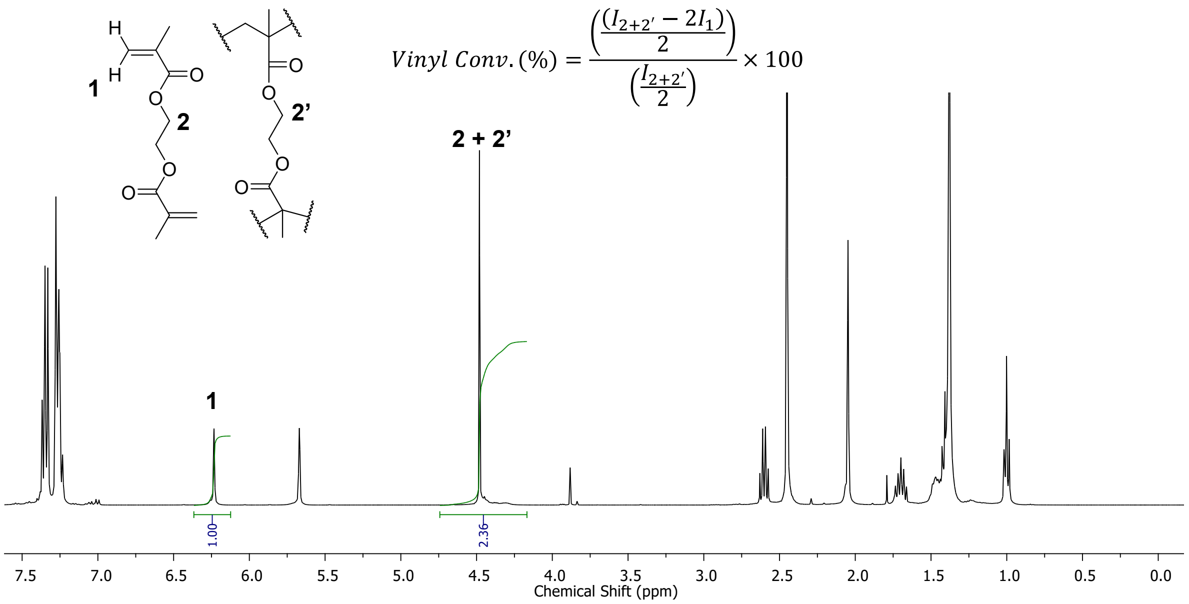

Figure S8 Exemplary determination of vinyl group conversion *via* ^{1}H NMR spectroscopy. The equation provided was used to determine vinyl group conversion (15%).

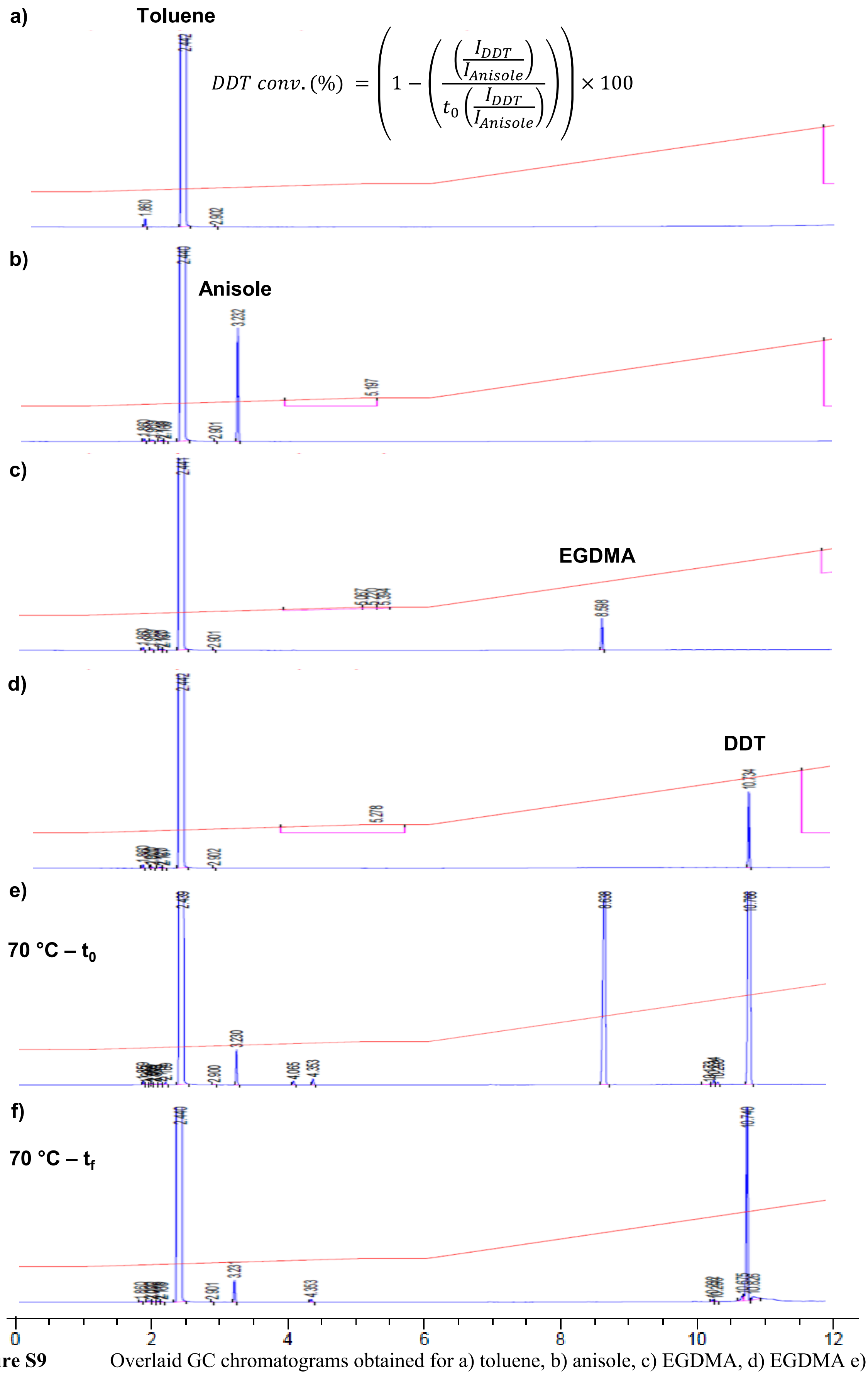

$$DDT\ conv.(\%) = \left(1 - \left(\frac{\left(\frac{I_{DDT}}{I_{Anisole}}\right)}{t_0\left(\frac{I_{DDT}}{I_{Anisole}}\right)}\right)\right) \times 100$$

Figure S9 Overlaid GC chromatograms obtained for a) toluene, b) anisole, c) EGDMA, d) EGDMA e) an exemplary TBRT reaction mixture prior to thermal initiation and e) and f) the same reaction mixture after 24 hours at 70 °C. The equation provided was used to determine both telogen and EGDMA conversion.

Table S3 Kinetic data from the TBRT of EGDMA and DDT ($[EGDMA]_0/[DDT]_0 = 0.85$) at 70 °C.

		^{1}H NMR ($CDCl_3$)[a]			GC[b]		
**Temp. (°C)**	**Time (mins)**	**Vinyl conv. (%)**	**ln[M]0/[M]**	**LOG(1-α)**	**EGDMA Conv. (%)**	**Telogen Conv. (%)**	**Log([Tel]/[Tel]0)**
70°C	0	0	0.000	0.000	0	0	0.000
	3	0	0.000	0.000	0	1	0.006
	6	10	0.109	0.047	22	3	0.013
	9	15	0.166	0.072	28	3	0.015
	12	19	0.203	0.088	33	4	0.017
	15	23	0.263	0.114	38	6	0.029
	20	28	0.333	0.144	47	9	0.040
	25	34	0.409	0.178	54	11	0.051
	30	38	0.476	0.207	61	14	0.063
	35	43	0.562	0.244	67	16	0.074
	40	48	0.650	0.282	73	19	0.090
	50	57	0.846	0.367	83	23	0.116
	60	66	1.076	0.467	90	28	0.145
	75	79	1.551	0.674	96	37	0.202
	90	89	2.235	0.971	99	44	0.257
	105	98	3.922	1.703	<99	52	0.322
	120	>99	6.908	3.000	<99	54	0.335

[a] Determined by ^{1}H NMR analysis of the reaction mixture at the stated time intervals. [a] Determined by GC analysis of the reaction mixture at the stated time intervals.

Table S4 Kinetic data from the TBRT of EGDMA and DDT ($[EGDMA]_0/[DDT]_0 = 0.85$) at 80 °C.

		^{1}H NMR ($CDCl_3$)[a]			GC[b]		
**Temp. (°C)**	**Time (mins)**	**Vinyl conv. (%)**	**ln[M]0/[M]**	**LOG(1-α)**	**EGDMA Conv. (%)**	**Telogen Conv. (%)**	**Log([Tel]/[Tel]0)**
80°C	0	0	0.000	0.000	0	0	0.000
	3	5	0.053	0.023	15	0	0.002
	6	14	0.149	0.064	23	1	0.005
	9	21	0.240	0.104	34	6	0.025
	12	26	0.304	0.132	45	10	0.045
	15	34	0.422	0.183	55	13	0.058
	20	46	0.618	0.268	70	19	0.092
	25	58	0.877	0.381	81	25	0.126
	30	68	1.143	0.496	89	31	0.164
	35	76	1.423	0.618	94	37	0.199
	40	83	1.778	0.772	98	41	0.230
	50	92	2.513	1.092	<99	51	0.308
	60	<99	6.908	3.000	<99	56	0.357
	75	<99	6.908	3.000	<99	56	0.367
	90	<99	6.908	3.000	<99	56	0.364
	105	<99	6.908	3.000	<99	56	0.367
	120	<99	6.908	3.000	<99	56	0.363

[a] Determined by ^{1}H NMR analysis of the reaction mixture at the stated time intervals. [a] Determined by GC analysis of the reaction mixture at the stated time intervals.

Table S5 Kinetic data from the TBRT of EGDMA and DDT ($[EGDMA]_0/[DDT]_0 = 0.85$) at 90 °C.

Temp. (°C)	Time (mins)	^{1}H NMR ($CDCl_3$)[a]			GC[b]		
		Vinyl conv. (%)	ln[M]$_0$/[M]	LOG(1-α)	EGDMA Conv. (%)	Telogen Conv. (%)	Log([Tel]/[Tel]$_0$)
	0	0	0.00	0.000	0	0	0.000
	3	7	0.067	0.029	11	0	0.002
	6	19	0.207	0.090	36	7	0.033
	9	38	0.480	0.208	63	15	0.069
	12	58	0.872	0.379	80	25	0.125
	15	72	1.262	0.548	92	35	0.184
	20	90	2.273	0.987	99	48	0.281
	25	99	4.343	1.886	>99	57	0.365
90°C	30	>99	6.908	3.000	>99	57	0.362
	35	>99	6.908	3.00	>99	57	0.362
	40	>99	6.908	3.000	>99	57	0.362
	50	>99	6.908	3.000	>99	57	0.362
	60	>99	6.908	3.000	>99	57	0.362
	75	>99	6.908	3.000	>99	57	0.362
	90	>99	6.908	3.000	>99	57	0.362
	105	>99	6.908	3.000	>99	57	0.362
	120	>99	6.908	3.000	>99	57	0.362

[a] Determined by ^{1}H NMR analysis of the reaction mixture at the stated time intervals. [a] Determined by GC analysis of the reaction mixture at the stated time intervals.

Table S6 Kinetic data from the TBRT of EGDMA and DDT ($[EGDMA]_0/[DDT]_0 = 0.85$) at 100 °C.

Temp. (°C)	Time (mins)	^{1}H NMR ($CDCl_3$)[a]			GC[b]		
		Vinyl conv. (%)	ln[M]$_0$/[M]	LOG(1-α)	EGDMA Conv. (%)	Telogen Conv. (%)	Log([Tel]/[Tel]$_0$)
	0	0	0.00	0.000	0	0	0.000
	3	7	0.072	0.0315	9	-[C]	-[C]
	6	31	0.365	0.158	52	12	0.057
	9	79	1.566	0.680	93	37	0.204
	12	95	2.976	1.292	>99	53	0.324
	15	>99	5.521	2.398	>99	57	0.368
	20	>99	6.908	3.000	>99	59	0.392
	25	>99	6.908	3.000	>99	59	0.392
100°C	30	>99	6.908	3.000	>99	59	0.392
	35	>99	6.908	3.000	>99	59	0.392
	40	>99	6.908	3.000	>99	59	0.392
	50	>99	6.908	3.000	>99	59	0.392
	60	>99	6.908	3.000	>99	59	0.392
	75	>99	6.908	3.000	>99	59	0.392
	90	>99	6.908	3.000	>99	59	0.392
	105	>99	6.908	3.000	>99	59	0.392
	120	>99	6.908	3.000	>99	59	0.392

[a] Determined by ^{1}H NMR analysis of the reaction mixture at the stated time intervals. [a] Determined by GC analysis of the reaction mixture at the stated time intervals. [C]Reliable GC integrals could not be obtained for accurate determination of telogen conversion.

Figure S10 Exemplary ^{1}H NMR spectrum obtained from a TBRT reaction mixture after 24 hours. The equation provided was used to determine vinyl group conversion of >99%.

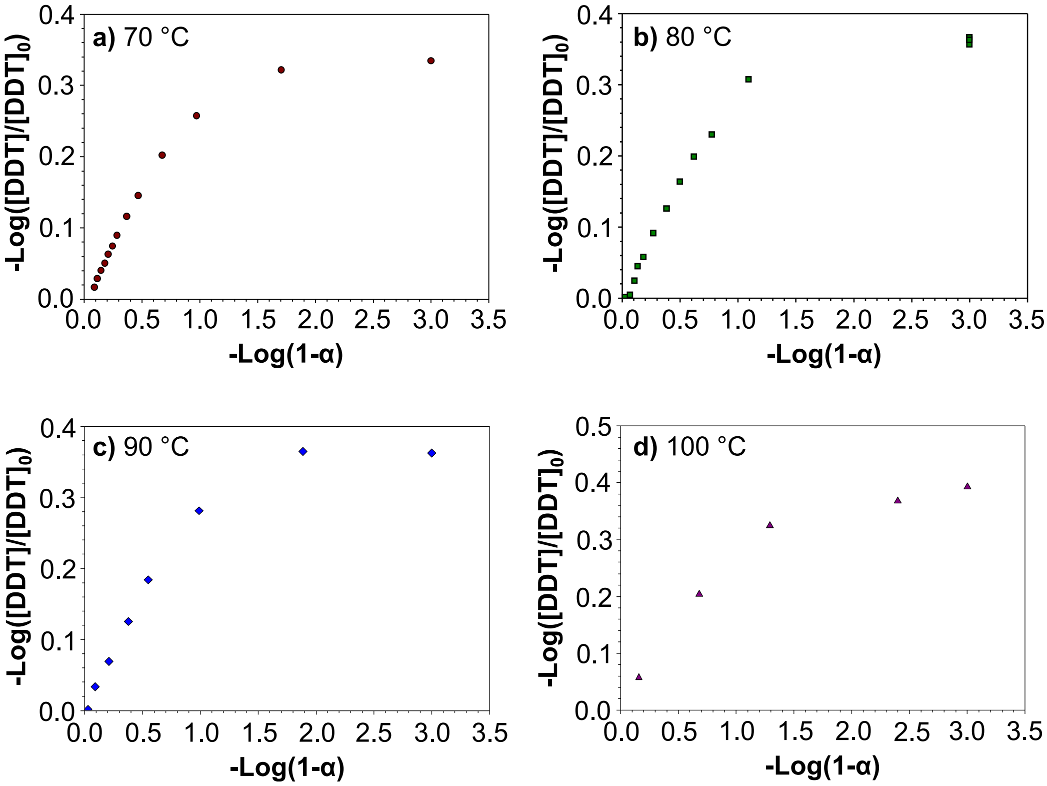

Figure S11 Full plots of -Log([Tel]/[Tel]$_0$) against -Log(1-α) obtained for TBRTs of EGDMA with DDT at a) 70, b) 80, c) 90 and d) 100 °C.

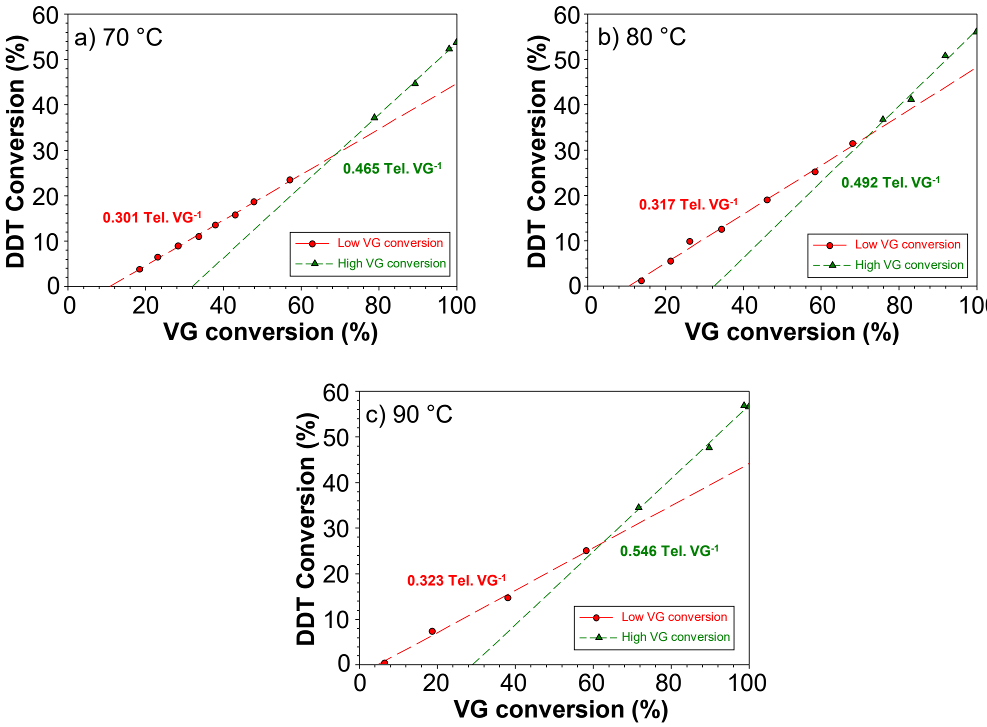

Figure S12 Gradient analysis of vinyl group and telogen (Tel.) conversion plots to determine relative telogen consumption during the early (0-80, red circles) and latter (>80%, green triangles) stages of TBRT. Note: $[EGDMA]_0/[DDT]_0 = 0.85$.

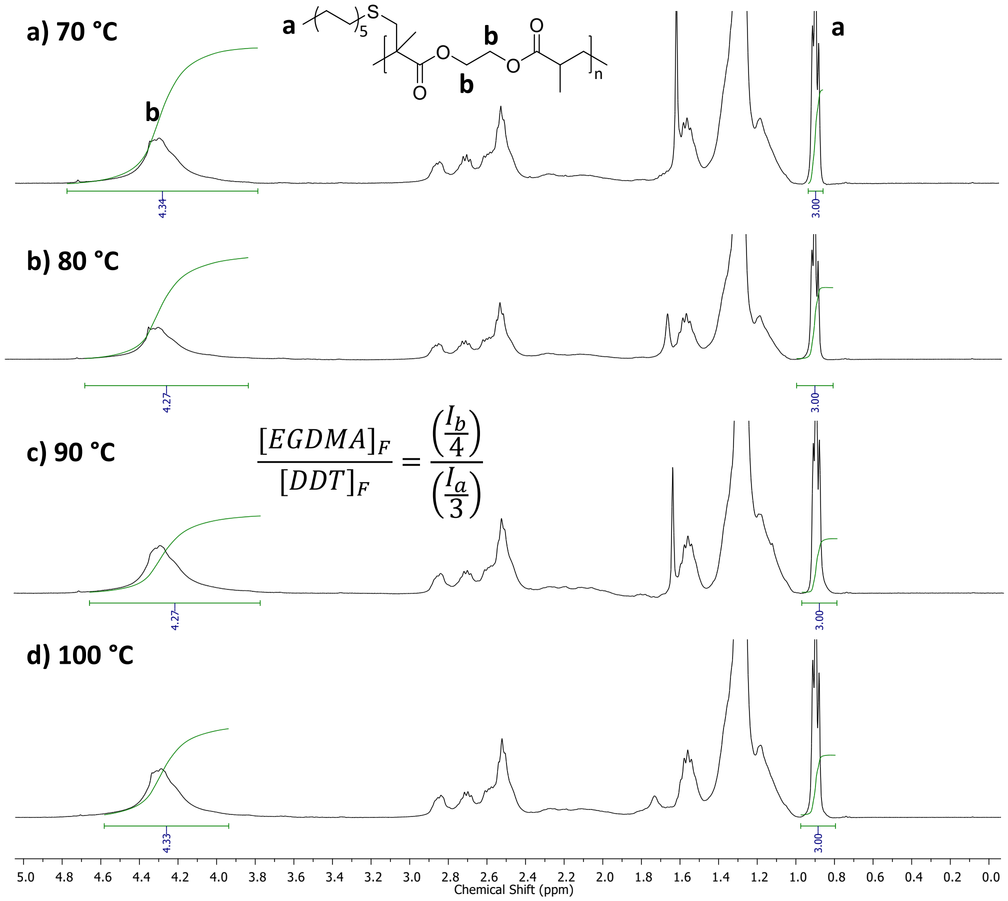

Figure S13 Overlaid ^{1}H NMR spectra of the highest molecular weight *p*(DDT-EGDMA) generated at a) 70°C, b) 80°C, c) 90°C and d) 100 °C.

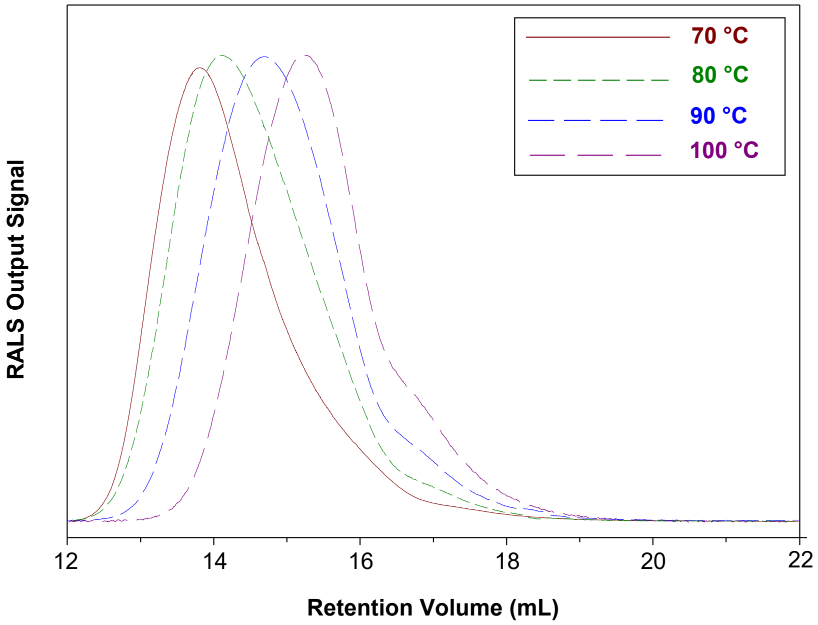

Figure S14 Overlaid RALS chromatograms obtained for *p*(DDT-EGDMA) generated *via* TBRT at 70°C (dark red solid line), 80°C (green short-dashed line), 90°C (blue medium-dashed line) and 100 °C (purple long-dashed line) using an $[EGDMA]_0/[DDT]_0$ ratio of 0.85.

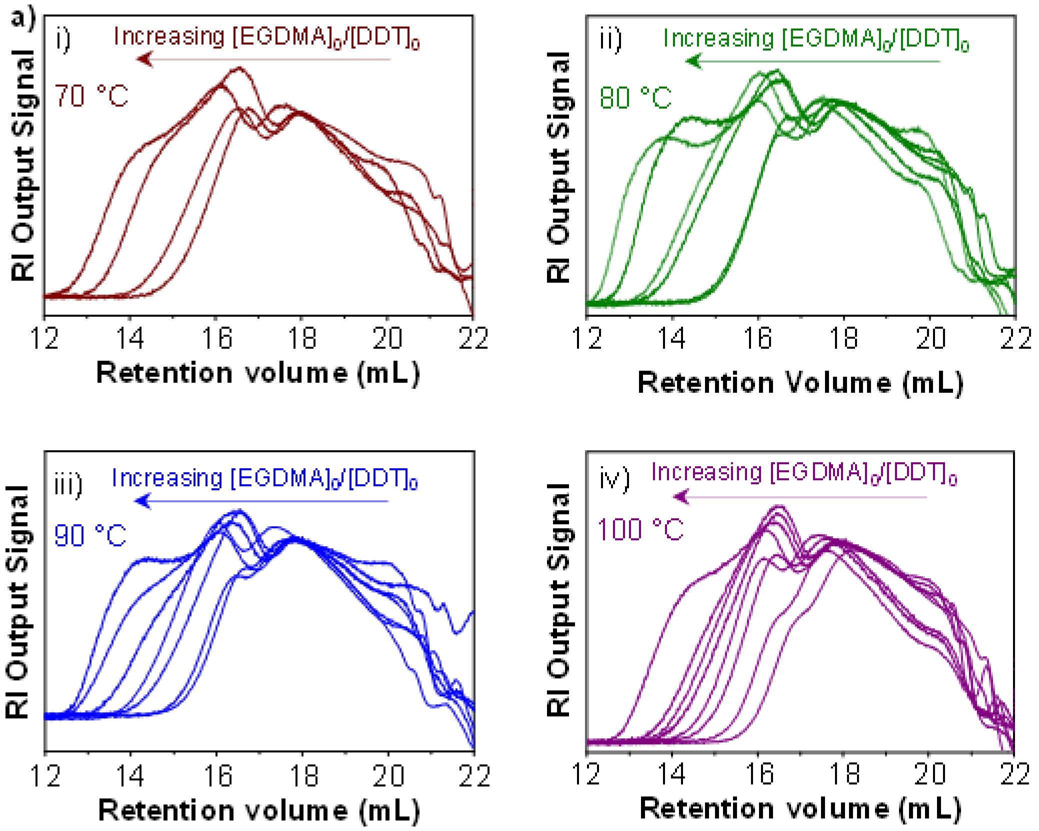

Figure S15 Overlaid a) RI and b) RALS chromatograms obtained from TD-SEC analysis of *p*(DDT-EGDMA branched polymers generated at varied $[EGDMA]_0/[DDT]_0$ ratios. i) 70°C (dark red, $[EGDMA]_0/[DDT]_0$ ratios: 0.75, 0.80, 0.83 and 0.85.), ii) 80°C (green, $[EGDMA]_0/[DDT]_0$ ratios: 0.75, 0.80, 0.83, 0.85, 0.88 and 0.90.), iii) 90°C (blue, $[EGDMA]_0/[DDT]_0$ ratios: 0.75, 0.80, 0.83, 0.85, 0.88, 0.90 and 0.93.) and iv) 100 °C (purple, $[EGDMA]_0/[DDT]_0$ ratios: 0.75, 0.80, 0.83, 0.85, 0.88, 0.90, 0.93 and 0.95).

Figure S15 ***(cont.)*** Overlaid a) RI and b) RALS chromatograms obtained from TD-SEC analysis of *p*(DDT-EGDMA branched polymers generated at varied $[EGDMA]_0/[DDT]_0$ ratios. i) 70°C (dark red, $[EGDMA]_0/[DDT]_0$ ratios: 0.75, 0.80, 0.83 and 0.85.), ii) 80°C (green, $[EGDMA]_0/[DDT]_0$ ratios: 0.75, 0.80, 0.83, 0.85, 0.88 and 0.90.), iii) 90°C (blue, $[EGDMA]_0/[DDT]_0$ ratios: 0.75, 0.80, 0.83, 0.85, 0.88, 0.90 and 0.93.) and iv) 100 °C (purple, $[EGDMA]_0/[DDT]_0$ ratios: 0.75, 0.80, 0.83, 0.85, 0.88, 0.90, 0.93 and 0.95).

Figure S16 Overlaid DSC thermograms obtained from *p*(DDT-EGDMA) generated by TBRT at a) 70°C (dark red), b) 80°C (green), c) 90°C (blue) and d) 100 °C (purple).

4. References

[1] R. A. Gregg, D. M. Alderman and F. R. Mayo, *J. Am. Chem. Soc.*, 1948, **70**, 3740–3743.
